# Genome and secretome of *Chondrostereum purpureum* correspond to saprotrophic and phytopathogenic life styles

**DOI:** 10.1371/journal.pone.0212769

**Published:** 2019-03-01

**Authors:** Rocio Reina, Harald Kellner, Jaqueline Hess, Nico Jehmlich, Immaculada García-Romera, Elisabet Aranda, Martin Hofrichter, Christiane Liers

**Affiliations:** 1 Department of Soil Microbiology and Symbiotic Systems, Consejo Superior de Investigaciones Científicas, Estación Experimental del Zaidín, Granada, Spain; 2 Unit of Environmental Biotechnology, Dresden University of Technology, International Institute Zittau, Zittau, Germany; 3 Department of Botany and Biodiversity Research, University of Vienna, Vienna, Austria; 4 Department of Molecular Systems Biology, Helmholtz-Centre for Environmental Research, Leipzig, Germany; USDA Forest Service, UNITED STATES

## Abstract

The basidiomycete *Chondrostereum purpureum* (Silverleaf fungus) is a saprotroph and plant pathogen commercially used for combatting forest “weed” trees in vegetation management. However, little is known about its lignocellulose-degrading capabilities and the enzymatic machinery that is responsible for the degradative potential, and it is not yet clear to which group of wood-rot fungi it actually belongs. Here, we sequenced and analyzed the draft genome of *C*. *purpureum* (41.2 Mbp) and performed a quantitative proteomic approach during growth in submerged and solid-state cultures based on soybean meal suspension or containing beech wood supplemented with phenol-rich olive mill residues, respectively. The fungus harbors characteristic lignocellulolytic hydrolases (GH6 and GH7) and oxidoreductases (e.g. laccase, heme peroxidases). High abundance of some of these genes (e.g. 45 laccases, nine GH7) can be explained by gene expansion, e.g. identified for the laccase orthogroup ORTHOMCL11 that exhibits a total of 18 lineage-specific duplications. Other expanded genes families encode for proteins more related to a pathogenic lifestyle (e.g. protease and cytochrome P450s). The fungus responds to the presence of complex growth substrates (lignocellulose, phenolic residues) by the secretion of most of these lignocellulolytic and lignin-modifying enzymes (e.g. alcohol and aryl alcohol oxidases, laccases, GH6, GH7). Based on the genetic and enzymatic constitution, we consider the ‘marasmioid’ fungus *C*. *purpureum* as a ‘phytopathogenic’ white-rot fungus (WRF) that possesses a complex extracellular enzyme machinery to accomplish efficient lignocellulose degradation during both saprotrophic and phytopathogenic life phases.

## Introduction

Wood-degrading organisms play an important role in carbon and nitrogen cycling. The most efficient ones are filamentous fungi, which can ecologically be categorized into brown-rot, white-rot and soft-rot fungi, colonizing compact wood (logs, branches, stumps) and degrading all polymeric cell wall components [1, 2]. Ascomycetous soft-rot fungi (SRF) erode the secondary wall or form discrete cavities within the cell wall where they break down mainly cellulose and hemicelluloses but have little or no effects on the wood lignin and the middle lamellae [3, 4]. Basidiomycetous brown-rot fungi (BRF) degrade primarily the polysaccharide components of wood and leave a partially modified lignin framework behind, whereas basidiomycetous white-rot fungi (WRF) decompose efficiently all cell wall components. The rate and extent of lignin, cellulose, and hemicellulose removal varies among white-rot species [5, 6]. They are considered to decompose the three wood components *via* the synergistic action of extracellular hydrolases and oxidoreductases classified within the CAZy database [7]. The class of Agaricomycetes includes numerous well-known WRF (wood and litter decomposers) and BRF, e.g. within the orders Polyporales (*Trametes versicolor*, *Phlebia radiata*, *Bjerkandera adusta*, *Irpex lacteus*), Gloeophyllales (*Gloeophyllum trabeum*) and Agaricales (*Agaricus bisporus*, *Stropharia coronilla*, *Agrocybe praecox*) [7].

Despite the crustous morphology of its fruiting body (basidiocarp), *Chondrostereum purpureum* (Pers. ex Fr.) Pouzar, belongs to the order Agaricales. It is a wood-decomposing fungus commonly found on broad-leaved trees in temperate and boreal vegetation zones. The fungus is saprotrophic mainly during the initial decomposition phase affecting felled trees or stumps. However, it also occurs as a secondary parasite causing the silverleaf disease in orchard trees [8, 9]. Spores can penetrate dead wood of living trees or on wounded wood where they grow and pair with other spores forming heterokaryotic mycelia that grows deeper into the wood [10]. When the hyphae of *C*. *purpureum* spread within a stump, tree vessels are occluded [11]. Induced dehydration combined with fungal toxins (sterpurenes, sesquiterpene metabolites) strengthens the adverse effects of the fungus in preventing the resprouting of stumps [11, 12]. The fungus consumes carbohydrates and during that process, it also decomposes lignin with the help of a large set of extracellular enzymes [1, 13–15]. When the decay process has penetrated deep enough into the wood, the ability of a stump to produce new sprouts diminishes and the stump dies. Later, *C*. *purpureum* is quickly replaced by other fungi [16]. Due to these abilities, *C*. *purpureum* has been used as a biocontrol agent against sprouting and root suckering of tree species such as red alder (*Alnus rubra*), black cherry (*Prunus serotina*), white birch (*Betula papyrifera*) and aspen (*Populus* spp.) [9, 17, 18].

Fruiting bodies of *C*. *purpureum* have a characteristic laminar-like shape and therefore, it was for a long time taxonomically assigned within the Polyporales (Meruliaceae; [18]). Matheny et al. [19] analyzed a larger phylogeny of agaric species, which resulted in the affiliation of *C*. *purpureum* within the Agaricales order, more precisely in the family of Cyphellaceae within the ‘marasmioid clade’ (comprising seven fungal families). However, other recent reports have claimed no strict consensus about the phylogenetic position of *C*. *purpureum*, being still included either within Polyporales or Agaricales [9, 20].

Beside the discrepancy in the phylogenetic affiliation of *C*. *purpureum*, there is only little known concerning its lignocellulolytic enzyme machinery, by which the fungus accomplishes its saprotrophic life style with that strong tendency to pathogenicity. In this context, it is yet not clear to which type of wood-rot fungi *C*. *purpureum* actually belongs. Next generation sequencing has enormously accelerated studies in fungal genomics and evolution, for example, the genomes of over 50 basidiomycetous fungi have been made available in 2014, including those of numerous Polyporales species [21]. Riley and coworkers [21] were able to separate white-rot (and white-rot like fungi) *vs*. brown-rot species based on the content of lignocellulose decomposing genes. Kohler et al. [22] found dramatic lignocellulose-related gene losses in brown-rot and ectomycorrhizal species compared to white-rot species, and Hess et al. [23] observed a similar tendency within the genus *Amanita*. Floudas et al. [24], and recently Nagy and coworkers [25], used comparative genomics to trace back the origins of lignocellulose decay capabilities. Sipos et al. [26] used a similar approach to elucidate the ‘signature’ of the pathogenicity in the genus *Armillaria* and found a large repertoire of plant cell wall degrading enzymes and pathogenicity factors, which are seemingly involved in the severe tree pathogenicity of several *Armillaria* species.

Several recent studies have evaluated the gene inventory of saprotrophic fungi often in combination with transcriptomic and secretomic analyses. Thus, the secretomes of lignocellulose-degrading fungi were studied when growing on different complex media often based on lignocellulosic materials [27]. Among them were basidiomycetous fungi causing typical white or brown-rot in deadwood (e.g. *Phanerochaete chrysosporium*, [28–30]; *Pleurotus ostreatus*, [27] or *Serpula lacrymans*, [2]; *Postia placenta*, [31]), or so far unclassified/unspecific types of wood-rot (e.g. *Schizophyllum commune*, [24]) as well as phytopathogenic wood-decay fungi (i.e. facultative parasites such as *Heterobasidion irregulare*, [32] or *Armillaria mellea*, [33]).

From the biotechnological perspective, wood-degrading fungi and their enzymes are promising tools for the bioconversion of natural lignocellulose-containing polymers into renewable resources and feedstocks, e.g. chemicals and biofuels [34]. In that context, the fermentation of unused lignocellulosic by-products or their extractives, given in large quantities by various industrial processes manufacturing agricultural or forestal products, offers a promising approach to convert cheap, underutilized materials (e.g. rape straw, grain or olive mill residues) into useful final goods (e.g. base chemicals, fibers or fertilizers). An important preparing step of such biotechnological fermentation processes is the biological pretreatment of lignocellulosic materials, which reduces substances inhibiting fermentation (i.e. toxic, persistent and protecting plant ingredients like lignin, suberin, tannins and phenolics) *via* their enzymatic degradation and transformation [34–36]. For example, dry olive mill residue (DOR), a by-product of a two-phase extraction process during olive oil manufacturing, is rich in organic matter and nutritionally relevant substances, which makes it attractive for an agronomic use. However, DOR contains significant amounts of phytotoxic ingredients. For that reason, fungal pretreatments of DOR to get rid of the toxic compounds have been intensively studied during the last years [37]. It was demonstrated that fungus-treated DOR enhances the growth of tomato plants and hence is applicable as a valuable organic fertilizer [38–40].

From the eco-physiological point of view, the addition of agricultural by-products to fungal cultures may reflect growth conditions in a complex environment close to nature and could therefore stimulate the secretion of enzymes required for degradation, ‘digestion’ and detoxification of lignocelluloses [38, 41, 42]. On the other hand, to our best knowledge, not much is known about the effects of natural phenolics, tannins and humic substances in leaf-litter, organic soil fractions, compost or agricultural wastes (e.g. DOR) on the protein expression profiles of fungi [43, 44].

Against this ecological and biotechnological background, it has been our intention–besides the general analysis of the *C*. *purpureum* genome–to analyze the secretomes of this fungus when growing on natural substrates (beech and birch wood), on the agricultural by-product DOR and on nutrient-rich soy medium. This approach may both help to deepen our understanding of the physiology and ecology of *C*. *purpureum* and to develop new fungus-based technologies.

## Materials and methods

### Dry olive mill residue (DOR)

DOR was obtained from the Sierra Sur olive oil company in Granada, Spain (2011–2012 harvest). It was sieved, autoclaved in three cycles and stored at 4°C before use. The aqueous extract of DOR (abbreviated as ADOR) was produced by a 1:2 (w:v) DOR:water extraction process lasting 8 h under orbital shaking (170 rpm) and subsequent centrifugation and filtration through glass fiber filters (Whatman no. 5) [41].

### Fungal cultivation

The *C*. *purpureum* strain used was obtained from the German Collection of Microorganisms and Cell Cultures (DSMZ, Braunschweig, Germany) where it is deposited under DSM 4894. Pre-cultures were incubated at 24°C on 2% malt extract agar (MEA) over two weeks to obtain fresh inoculum. Solid-state fermentations (SFFs) with the fungus were performed in 250-mL Erlenmeyer flasks. Each flask contained 4 g of beech wood (BW) and 14 mL of distilled water. After autoclaving BW two times for 20 min at 121°C, the sterile wood was inoculated with 9 mL of a homogenized suspension from four fully overgrown agar plates in 80 mL sterile tap water. After the fungal mycelium had grown for one week at 25°C, half of the flasks were mixed with DOR (50% w:w) for preparing the DOR supplemented beech wood cultures (BWD). Sampling occurred weekly over an incubation time of seven weeks. To obtain the extracellular enzymes as well as the complete secreted protein profile, the harvested cultures were extracted with distilled water (1:5 w:v) by shaking on a rotatory shaker at 150 rpm for two hours. Extracts for label-free proteome analyses were prepared after seven weeks of cultivation by aqueous extraction, centrifugation and concentration *via* lyophilization.

To identify differences in the enzyme secretion pattern in dependence of the composition of the liquid media, submerged fermentation (SF) with *C*. *purpureum* was performed in 500 mL round-bottomed flasks using either 200 mL of complex soybean meal suspension (SM) or 200 mL of synthetic Kirk-medium (KM); the latter was prepared as described by [1]. SM was prepared with distilled water in a 3% ratio (w:v). To both culture media, ADOR (5% (v:v); ASKM & ASSM) as well as birch wood (1:2 (w:v); BSKM & BSSM) were supplemented after four days of fungal growth, respectively (Table 1).

**Table 1:** Composition of solid and liquid media used for the analysis of the proteomes of *Chondrostereum purpureum*.

Medium	Composition
Solid state fermentation (SSF)	
BW	beech wood (1:3 with distilled water, w:v),
BWD	beech wood (1:3 with distilled water, w:v), plus DOR (50%, w:w)
Submerged fermentation (SF)	
KM	Kirk-medium
SM	Soybean meal medium (3% suspension in distilled water, w:v),
ASKM	KM, plus ADOR (5%, v:v)
ASSM	SM, plus ADOR (5%, v:v)
BSKM	KM, plus birch wood (1:2, w:v)
BSSM	SM, plus birch wood (1:2, w:v)

The liquid cultures were incubated at 25°C and 100 rpm on a rotatory shaker. Every second or third day, samples were taken (1.5 mL) from the culture liquids until the end of the experiment on day 16 and used for enzymatic measurements. After the end of the experiment, culture liquids were concentrated by ultrafiltration, lyophilized and used for label-free proteome analyses. All treatments were carried out in triplicate.

### Enzymatic activity measurements

Manganese peroxidase (MnP) activity was determined as described by [40] by following the formation of Mn^3+^-malonate complexes (Ɛ_270_ = 11.95 mM^-1^ cm^-1^) spectrophotometrically in the presence of MnCl_2_ (0.5 mM) and H_2_O_2_ (0.1 mM). To distinguish between activities of laccase and manganese-independent peroxidase (MiP, including lignin, versatile and generic peroxidase) a sequential assay was performed that based on the oxidation of ABTS (0.3 mM; ε_420_ = 36 cm^-1^ mM^-1^) with and without H_2_O_2_ addition (0.1 mM); peroxidase activity was corrected by the calculated Lac activity [45–47]. Unspecific peroxygenase activity (UPO) was measured as reported by Ullrich et al. [48] using veratryl alcohol at 310 nm (Ɛ_310_ = 9.3 mM^-1^ cm^-1^) and pH 7.0 in the presence of 1 mM H_2_O_2_. The mean of triplicate measurements was calculated and expressed in international units (U). An international unit is defined as the amount of enzyme that forms 1 μmol of product or converts 1 μmol of substrate per minute under assay conditions. Enzymatic activities detected in SSF cultures were expressed in U g^-1^ and those determined in SF cultures as U L^-1^.

### DNA isolation and genome sequencing

Genomic DNA was purified from a dikaryotic strain of *C*. *purpureum*. High quality RNA-free DNA was obtained using DNeasy Plant Maxi Kit (QIAGEN). The obtained gDNA (a total of 1 μg) was fragmented with Ion Shear Plus Reagent to obtain a 200-basepair-read library. The reaction was performed at 37°C during 4 min. Fragmented gDNA was purified with the Agencourt AMPure XP Kit and the fragment size was checked with the Agilent 2100 Bioanalyzer. Adapters were ligated and blunt-end was nick-repaired with the Ion Plus Fragment Library Kit, then the ligated DNA was again purified. Subsequently, fragments of 250 bp were size-selected on an E-Gel SizeSelect agarose gel. Fragment size was again checked using the Agilent 2100 Bioanalyzer and a PCR amplification was not required. Library was diluted to a final concentration of 26 pM with the aid of Bioanalyzer to calculate the dilution factor. Template-positive ISPs containing clonally amplified DNA fragments were obtained using the Ion OneTouch 200 Template Kit v2 according to the manufacturer protocol. The quality of the unenriched template-positive ISPs was assessed using a Qubit 2.0 fluorometer and the Quality Control of the Ion Sphere kit. The percentage of Templated ISPs was 16%. ISP enrichment was performed with the aid of Ion OneTouch ES. The enriched template-positive ISPs were sequenced using an Ion Torrent Personal Genome Machine (PGM) (Life Technologies; Grand Island, NY, USA) according to the manufacturer's protocols provided for a 318v2 chip.

### Assembly and genome annotation

All reads obtained from polyclonal and low quality ISPs were excluded. The assembly was performed according to the procedure described by Kellner et al. [49]. Only raw-reads with a read length between 120–250 bp were considered. They were assembled using MIRA 4 [50] with an accurate sensibility and a minimum of 50 reads per contig. The obtained contigs were re-assembled with the Geneious R8 *de novo* assembler to filter for duplicate contigs. To assess the completeness of the assembled genome, we used CEGMA v2.5 [51] and the quality of the assembly was calculated using QUAST [52]. To verify the assembly quality and to calculate the coverage (empirical: number of reads * read length / assembly size), a remapping approach was performed. All reads were mapped against the assembly using the Geneious assembler (sensitivity: low/fastest) to analyze coverage and their uniformity.

*Ab initio* gene prediction was performed with Augustus [53], using *Laccaria bicolor* as a reference organism. No transcript variants were selected. The contigs with higher coverage after remapping were chosen for searching the rRNA genes’ cistron. The functional annotation of the protein-coding genes after prediction with AUGUSTUS was carried out by a bulk blastp search against a non-redundant database (nr) obtained from GenBank. The output file (xml) with the BLAST results was imported into the Blast2GO platform, which was used for creating a *C*. *purpureum* gene database as well as for mapping and annotating of the BLAST results and displaying gene ontology terms (GO), which were merged with InterPro motifs after an InterProScan. The latter step helped in functional annotation of the genome and finding of relevant proteins in the total set of 13,739 predicted gene models. Furthermore, it provided the annotated protein database for subsequent proteomic analyses. The sequences of interest, such as unspecific peroxygenase (UPO), dye-decolorizing peroxidase (DyP), laccase (Lac) and class II peroxidase (POD), were also identified and annotated manually by BLAST searches against the created proteine database and the genome contigs using gene models as references. A comprehensive analysis of CAZy genes was performed by custom BLAST searches and by using the dbCAN webserver (settings: HMMER search, E-Value <1e-4, coverage >0.3) [54]. The raw data and genome assembly is accessible at National Center for Biotechnology Information under LBLO00000000 (BioProject PRJNA281625).

### Phylogenetic analysis

To confirm broad phylogenetic placement of *C*. *purpureum* using genome-scale data, 35 published Agaricomycete genomes [21, 26, 44, 55–60] as well as the newly sequenced *C*. *purpureum* were used to infer a species tree. To this end, predicted proteomes of all species were clustered into gene families using the FastOrtho (http://enews.patricbrc.org/fastortho/) implementation of the OrthoMCL software [61] with default search and clustering parameters. This resulted in a dataset of 43,749 clusters of which 1,368 were single copy and present in at least 30 species, and consequently, selected for phylogenetic analysis. Amino acid (aa) sequences constituting each cluster were aligned using Canopy (https://github.com/chunxiangli/Canopy) with the PRANK aligner [62] and three iterations of alignment and guide tree building. The resulting alignments were trimmed to remove poorly aligning regions using the software TrimAl [63] with the ‘-automated1’ algorithm. ParGenes [64] was used to infer the best evolutionary model and a gene tree for each of the 1,368 clusters using the following parameters to RAxML-NG: ‘-s 10 -p 10 -b 100’ [65]. Genes with strong phylogenetic signal were identified based on average bootstrap support (BS) of >50% and a minimum alignment length of at least 150 aa.

This resulting high quality set of genes was then used to infer a species tree using two complementary approaches: i) a partitioned Maximum Likelihood approach, encompassing a total of 84,248 sites and ii) ASTRAL-III, a super tree approach based on the multispecies coalescence [66]. For i) PartitionFinder2 [67] was used to identify the best-fit partitioning scheme using linked branch lengths, AICc-based model selection, the rclusterf search algorithm and the following models of protein evolution: LG, LG+G, LG+I+G, WAG, WAG+G, WAG+I+G, JTT, JTT+G, JTT+I+G, LG4X, LG4M+G. RAxML-NG [65] was run using the partition model, encompassing 112 partitions, the Majority Rule Extended automatic stopping criterion for bootstrap trees and scaled branch lengths. To infer the coalescent species tree (ii), gene trees of the high quality gene set were modified to remove nodes with less than 70% BS and ASTRAL-III was run using default parameters.

### Evolutionary analyses of gene content

Genome-wide duplication and loss rates across the 36 studied genomes were inferred using CAFÉ v4.1 [68]. To this end, an ultrametric tree was estimated using R8s v1.81 [69], with the penalized likelihood algorithm and root age arbitrarily set to 350 MYA. As input tree, we used the topology of the ASTRAL tree but with branch lengths estimated as substitution per site using RAxML-NG with the partitioned model (see above). OrthoMCL clusters were filtered to remove clusters with more than 100 gene copies in any of the species, as well as families represented in less than five species. This resulted in a final dataset of 12,032 clusters with wide taxonomic distribution. CAFÉ was run estimating a single gain (λ) and loss (μ) parameter across the tree. Gene families with significantly slower or faster rates of evolution were identified using branch-specific P-values reported by CAFÉ with a threshold of 0.01.

For target gene families of interest (GH6, GH7 and Lac), we also implemented a full phylogenetic approach for inferring gene duplications and losses. Gene trees for each target cluster were aligned as above. TreeFix v.1.1.10 [70] was run for species-tree aware error correction of each gene tree using the following options: nquickiter = 100 and niter = 1000, and the best-fit model determined by ParGenes [64], if available and WAG+G otherwise. Corrected gene trees were then reconciled with the species tree using NOTUNG 2.9 [71] in the ‘phylogenomics’ mode.

Furthermore, a principal component analysis was performed for the number of core lignocellulolytic enzyme encoding genes (according to Riley et al. [21] using PAST 3.22 [72]).

### Proteome analysis of the *C*. *purpureum* strain

Lyophilized secreted protein fractions of fungal culture liquid (3 mg) were resuspended in 4 mL SDS buffer (1.25% SDS, 0.1 M TRIS, 0.3% DTT), a spatula of glass beads were added and the solution was incubated for one hour at room temperature under shaking. Afterwards, the FASTPrep (5.5 m/s, 45 sec, 3 cycles) protocol was applied followed by further steps including three cycles of freezing and thawing (freezing in liquid nitrogen, thawing in 60°C water bath), the addition of 0.6 mL of 10% (w/v) SDS solution and two cycles of ultrasonic treatment using an ultrasonic disintegrator (ultrasonic processor UP50H equipped with ultrasonic probe MS7, Hilscher Inc., Germany; 4°C, 2 min/80% amplitude/80% power, break of 2.5 min between cycles). Phenol extraction was applied to the sample supernatant by adding 4 mL phenol solution (10 g mL^-1^ in ddH_2_O) and incubating under shaking at 500 rpm at room temperature for one hour. The mixture was centrifuged at 4°C and 12,000 × *g* for 10 min to achieve phase separation (Sorvall RC 6 plus, Thermo Fisher Scientific, Waltham, MA, USA). The lower phase was mixed with a fivefold volume of ice cold 100 mM ammonium acetate in methanol. Precipitation was performed overnight at -20°C followed by centrifugation at 12,000 × *g*. The supernatant was removed and the pellet was air-dried. The resulting protein pellets were separated on SDS-PAGE with a 15°μL sample buffer. Each sample lane was then cut into one gel band and prepared for proteolytic cleavage. Protein lysate was reduced (2.5 mM DTT for one hour at 60°C) and alkylated (10 mM iodacetamide for 30 min at 37°C). Proteolysis was performed overnight using trypsin (Promega, Madison, WI, USA) with an enzyme/substrate ratio of 1:25 at 37°C. Peptide lysates were extracted from the gel and desalted using SOLAμ (Thermo Scientific) [73].

The peptide lysates were separated on a UHPLC system (Ultimate 3000, Dionex/Thermo Fisher Scientific, Idstein, Germany). Peptides were first trapped for 3 min on a C18-reverse phase trapping column (Acclaim PepMap 100, 75 μm × 2 cm, particle size 3 μM, nanoViper, Thermo Fisher Scientific), followed by separation on a C18-reversed phase analytical column (Acclaim PepMap 100, 75 μm × 25 cm, particle size 3 μM, nanoViper, Thermo Fisher Scientific) using a two-step gradient (90 min from 4% to 30% B, then 30 min from 30% to 55% B; A: 0.1% formic acid in MS-grade water; B: 80% acetonitrile, 0.1% formic acid in MS-grade water) with a solvent flow-rate of 300 nL min^- 1^ and a column temperature of 35°C.

Mass spectrometry was performed on a Q Exactive HF mass spectrometer (Thermo Fisher Scientific, Waltham, MA, USA) with a TriVersa NanoMate (Advion, Ltd., Harlow, UK) source in LC chip coupling mode with the following settings: MS resolution 120,000, MS automatic gain control (AGC) target 3,000,000 ions, maximum injection time for MS 80 ms, intensity threshold for MS/MS of 17,000 ions, dynamic exclusion 30 sec, TopN = 20, isolation window 1.6 *m/z*, MS/MS resolution 15,000, MS/MS AGC target 50,000 ions, maximum injection time for MS/MS 120 ms.

Proteome Discoverer (v1.4.1.14, Thermo Scientific) was used for protein identification and the acquired MS/MS spectra were searched with the Sequest HT algorithm against the protein-coding database of *C*. *purpureum* (containing 13,739 protein-coding gene entries). Enzyme specificity was selected to trypsin with up to two missed cleavages allowed using 10 ppm peptide ion tolerance and 0.05 Da MS/MS tolerances. Oxidation (methionine) and carbamylation (lysine and arginine) were selected as variable modifications and carbamidomethylation (cysteine) as a static modification. Only peptides with a false discovery rate (FDR) <1% calculated by Percolator and peptide rank = 1 were considered as identified. Relative protein abundances were calculated based on the normalized spectral abundance factor (NSAF) [74].

### Statistical analysis

Secretome samples were triplicated for each treatment. Hotelling’s T2 was performed to find statistically significant differences between treatments and principal component analysis (PCA) using non-linear iterative partial least squares algorithms (NIPALS) was used to determine the main trend in the data set and to compare the samples replicates and treatments. The software used for statistical analyses were SPSS Statistics 21, Unscrambler X10.2 and R [75].

## Results

### Assembly and quality assessment of *Chondrostereum purpureum* draft genome

The genome of *C*. *purpureum* was assembled from 3,971,460 quality filtered reads obtained from the Ion Torrent PGM System. The average fragment size was 168 bp and a final 41.2 Mbp-sized draft genome organized in 3,435 contigs was obtained after the assemblies using MIRA and Geneious R8 (Table 2).

**Table 2:** Statistical assembly of the *C*. *purpureum* genome.

Assembly statistics	
Max contig length	207,970
Min contig length	390
Number of contigs	3,435
N50	23,869
L50	655
N's per 100 kbp	98
Annotation statistics	
Number of predicted CDS	13,739
Maximal CDS length (bp)	15,468
Mean CDS length	1,522
GC content (%)	47.5

A remapping approach resulted in a uniform coverage (S1 Fig), and the average coverage was 15.2x. From the subset of 248 ultra-conserved CEGMA CEGs considered, we found 229 (92.3%) complete and 237 (95.6%) partial proteins sequences. In sum, a number of 13,739 protein-coding gene models was predicted. For 99% of the sequences, a BLAST hit was obtained and 30%, i.e. 4,016 sequences of the gene models contained a GO term associated. Considering the ‘biological process domain’, 480 of the predicted models with a GO were related to the metabolism of *C*. *purpureum* (S2 Fig). An assignment of gene sequences to enzymes and their classification according to EC nomenclature is given in S3 Fig.

### Classification of the *C*. *purpureum* genome and proteome

**Fig 1:**
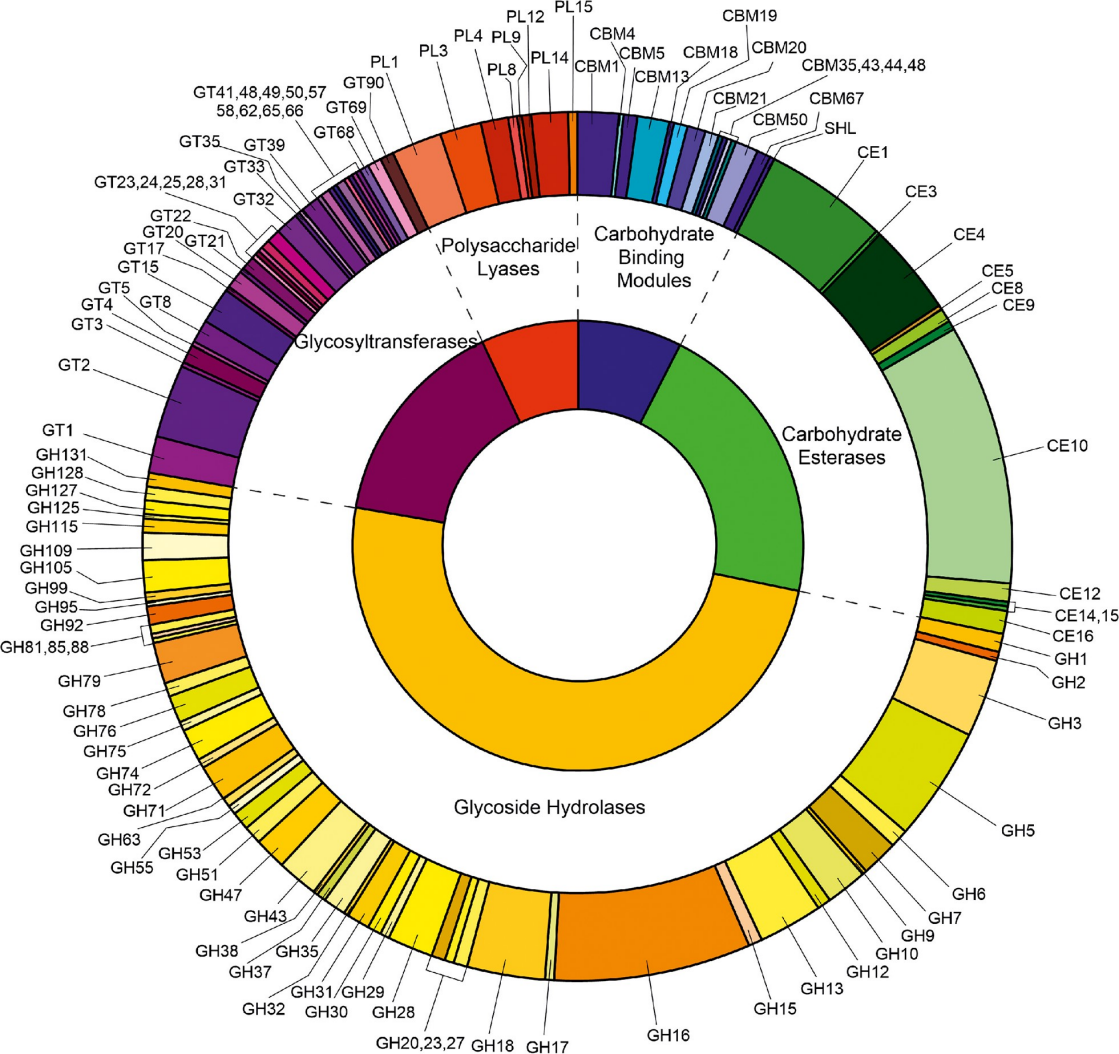
*C*. *purpureum* CAZy genes classification (except auxiliary activities of oxidoreductases from AA1 to AA9).

The *C*. *purpureum* genome comprised an extensive repertoire of CAZymes characteristic for wood-rot fungi, including hydrolytic enzymes attacking cellulose and hemicelluloses (e.g. almost 50% GHs, CEs & CBMs; Fig 1) as well as multiple oxidative ‘auxiliary’ activities acting on lignin or lignin-derived aromatics (e.g. class II heme peroxidase, Lac, DyP and UPO; S4 Fig).

**Fig 2:**
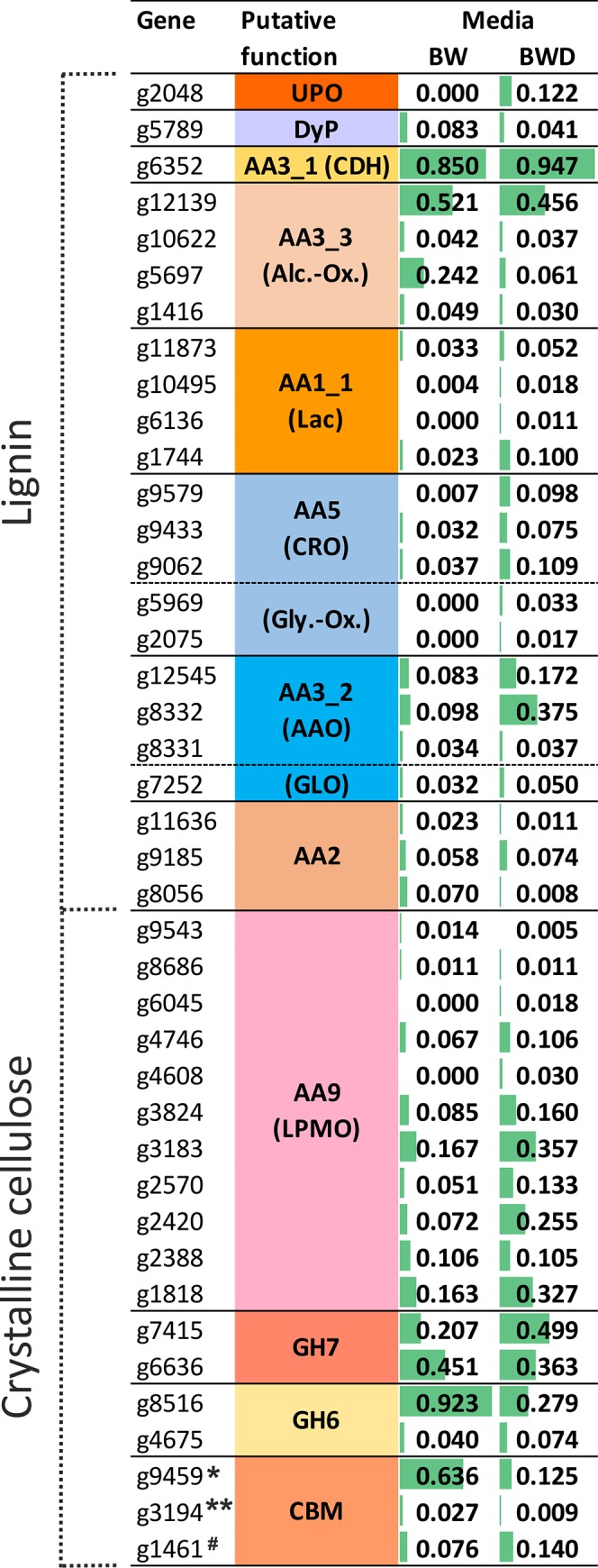
Distribution of the lignocellulose-degrading enzymes secreted by *C*. *purpureum* (after seven weeks of cultivation). Relative abundance (% NSAF) of the proteins found in solid state cultures containing beech-wood (BW) or beech-wood and DOR (BWD) is given (Table 1). CBM1 of *GH7, **GH131 (β-glucanase) and ^#^acetylxylan esterase.

**Fig 3:**
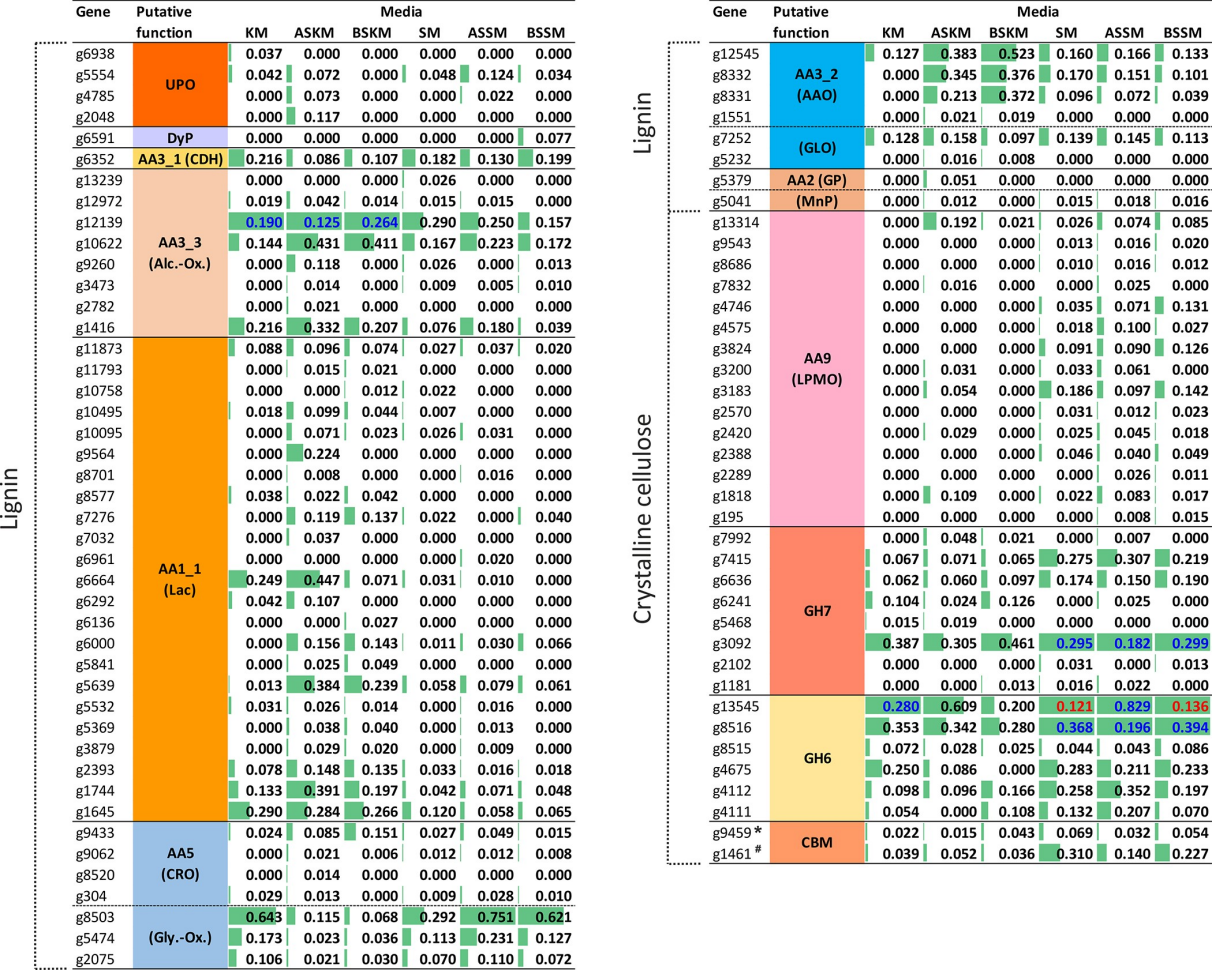
Distribution of the lignocellulose-degrading enzymes secreted by *C*. *purpureum* (after 16 days of cultivation). Relative abundance (% NSAF, >0.01) of the proteins found in liquid cultures of synthetic KM, ASKM, BSKM and complex SM, ASSM and BSSM (Table 1). To obtain an appropriate resolution, high NSAF values (>1%) were divided by the factor 10 (blue) or 100 (red). CBM1 of *GH7 and ^#^acetylxylan esterase.

Many of these proteins were secreted by the fungus during its growth both in synthetic and complex media (1,151 and 981 in solid-state and liquid media, respectively), especially key enzymes involved in lignocellulose decomposition (Figs 2 & 3). Overall, CAZy enzymes involved in lignocellulose degradation represented between 46 and 53% as well as 35 and 46% NSAF in the *C*. *purpureum* secretome on solid (BW and BWD, respectively) and liquid cultures (KM- and SM-based, respectively), respectively (S5 & S6 Figs). During liquid cultivation, distinct secretion patterns were observed (S7 Fig). Altogether 67 GHs, seven CEs, eight PLs, 28 AAs and ten CBMs showed significant differences between the different cultivations.

#### Glycoside Hydrolases

The analysis of the putative *C*. *purpureum* enzymes classified in the genome using dbCAN indicated that almost half of them were glycoside hydrolases (GHs, 49.5%) (Fig 1). The most abundant GHs were found in the functionally diverse families GH16 and GH5 with 43 and 25 sequences, respectively. In addition, a large set of genes from other families encoding cellulolytic (e.g. GH6, GH7, GH12), hemicellulolytic (e.g. GH10, GH11, GH30) and pectinolytic (e.g. GH43, GH28, GH53) proteins are present in the *C*. *purpureum* genome and most of them were expressed by the fungus during the different liquid and solid cultivations (GH6, 7, 10, 12, 28 & 43; S7 Fig). The GH class was with 34% and 32% NSAF and with a number of 112 and 124 different genes the most abundant group in the secretome obtained from the BW and BWD cultures of the fungus. Among them, GH6 and GH7 gene products (e.g. *g8516* & *g6636*, respectively) were strongly expressed (~0.9 & 0.5%, respectively) in the BW cultures compared to the other lignocellulolytic enzymes. In the liquid cultures, a high percentage of GHs (~44% NSAF of the overall set of secreted proteins) was determined in SM and BSSM compared to the other liquid media (23–29% NSAF). High relative abundances were specifically found for the GH6 class (e.g. *g13545*), with 12%, 8% and 14% NSAF in SM, ASSM and BSSM, respectively. Together with the gene product *g8516* (<3.9% NSAF), both were the most abundant proteins secreted by *C*. *purpureum* in all soybean-containing media (S8 Fig). The expressed CBM1 modules found in the fungal secretome pertain to a β-glucanase (GH131; *g3194*) and an exoglucanase (GH7; *g9459*); the latter occurred with a relatively high abundance mainly in BW (0.6% NSAF) (Fig 2).

#### Other CAZymes

CBMs are necessary for the functioning of most GHs; they were found to be present in the *C*. *purpureum* genome in form of 14 different families (e.g. CBM1, 13, 18, 67) and could be identified in the secretome especially in BW (~0.6% NSAF for *g9459*) and soybean-based media (0.1–0.3% NSAF for *g1461*).

Carbohydrate esterases (CE) accounted for approx. 22% of the predicted CAZy gene models with hydrolytic activities. Among them were putative pectinolytic enzymes like CE4, 8, 9 and 15, while the CE10 family (containing carboxyl and aryl esterases) was the most abundant one with 57 predicted genes. Some of the CE families were expressed at higher levels (e.g. CE4, 8, 9; S7 Fig) when the fungus was growing in liquid media containing either soybean, wood or ADOR components. An acetylxylan esterase belonging to the CE1 family associated with a CBM1 module (*g1461*) was expressed by the fungus in soybean and wood cultures (Fig 3).

The addition of phenolic DOR to the solid-state cultures seemingly stimulated the secretion of polysaccharide lyases (PL). The relative amount of PL was found to be three-fold higher (9.6–2.9% NSAF) in BWD than in BW (S5 Fig). Also non-CAZy hydrolases such as peptidases (25.4–11.9% S5 Fig) showed higher relative protein amounts after DOR addition. Interestingly, the secretion level of the peptidase enzyme subclass (EC 3.4.x) was also remarkably increased (up to 15% NSAF) when the fungus grew in ASSM compared to SM without ADOR supplementation (8% NSAF) (S6 Fig).

#### Oxidoreductases

Altogether 153 oxidoreductases with relevance for lignocellulose decomposition were found in the *C*. *purpureum* genome and 81 of them were expressed during the different cultivations (Table 3).

**Table 3:** Oxidoreductases found in the *C*. *purpureum* genome and expressed in fungal cultures.

Proteins	Total	Expressed ^a^
**UPO** ^b^	8	5
**DyP** ^b^	7	2
**AA1**	47	27
Lac	45	25
FeOx Fet3	1	1
Fungal pigment oxidase (MCO)	1	1
**AA2 class II peroxidase (POD)**	4	2
MnP	2	1
GP	2	1
**AA3 GMC**	36	16
**AA4 VAO**	3	0
**AA5 GLX and CRO**	12	9
**AA7 GOO**	3	3
**AA8 iron reductase domain**	2	0
**AA9 LPMO**	31	17

Abbreviations: unspecific peroxygenase (UPO), dye-decolorizing peroxidase (DyP), CAZy classified auxiliary activities (AA): AA2 class II peroxidases (manganese peroxidase, MnP and generic peroxidase, GP) as well as AA1 (laccase, Lac), ferroxidase (FeOx) and fungal pigment multicopper oxidase (MCO), AA3 (glucose-methanol-choline oxidoreductase, GMC), AA4 (vanillyl alcohol oxidase, VAO), AA5 (glyoxal oxidase, GLX) and cooper radical oxidase (CRO), AA7 (glucooligosaccharide oxidase, GOO), AA8 and AA9 (lytic polysaccharide monooxygenase, LPMO) enzymes.

^a^Number of expressed genes in the secretomes obtained from different culture media.^b^Non CAZy protein families.

Among them are typical enzymes involved in lignocellulose breakdown caused by wood-rot fungi. Besides AA1 (multicopper oxidases, e.g. Lac) and AA3 (GMC, e.g. cellobiose dehydrogenase or alcohol oxidase), a high number of AA genes were found to encode LPMO (AA9) and more than half of them were expressed with NSAF >0.01% (17 out of 31 LPMO genes). During growth on wood (BW and BWD), LPMO made up the largest number of proteins among the lignocellulose-modifying enzymes. Some of them had a higher relative abundance in media supplemented with phenol-rich olive mill residues (e.g. in BWD with 0.36, 0.26, 0.33% NSAF for *g3183*, *g2420*, *g1818*, respectively and in ASKM with 0.19, 0.05, 0.11% NSAF for *g13314*, *g3183*, *g1818*, respectively), or in soybean-based liquid media (Figs 2 & 3). Whereas none of them was present in synthetic KM without any additive, 15 genes encoding for LPMO were expressed in soybean-based medium (e.g. 0.097–0.186% NSAF for *g3183*) and seven of these LPMOs showed significant differences during cultivation (S7 Fig).

Four class II peroxidases were found in the genome of *C*. *purpureum* (S9 Fig). Among them two MnPs (*g2700* & *g5041*), the key enzyme type of incipient lignin degradation by white-rot fungi, were identified according to the conserved manganese-binding aa residues (D34, D39 & D179; S10 Fig). The presence of MnP was confirmed by respective activity measurements in both solid and liquid media (<10 to 30 U L^-1^; S9 and S10 Figs) and by detection of an expressed short MnP protein (*g5041*; Fig 3) mainly in soybean-based medium but also in ASKM. Due to the absence of typical manganese-binding aa residues and LiP-characteristic tryptophan (at position W171 in *P*. *chrysosporium*, [76]), the other two sequences (*g5379*, *g10149*) of class II peroxidases seemingly represent generic peroxidases (GPs) that typically oxidize phenolics. One of the GPs was found to be expressed exclusively in ASKM (S10 Fig).

In addition to class II peroxidases, sequences encoding for seven DyPs and eight HTPs/UPOs were predicted in the genome. Due to the presence of two specific amino acid motifs, PCP and EHD [77], six complete UPO sequences could be assigned to the ‘short’ UPOs. The other two sequences were incomplete but phylogenetic analysis related them also to the clade of ‘short’ UPOs. The fungus secreted one DyP (*g5789*) in BW and BWD, whereas one ‘short’ UPO (*g2048*) was exclusively found in BWD, the presence of which was confirmed by a corresponding enzymatic activity 0.5 U g^-1^ after six weeks of cultivation (Figs 2 & S11). In SF, another DyP gene (*g6591*) and four further ‘short’ UPO genes (*g4556*, *g4785*, *g5554*, *g6938*) were expressed by the fungus. UPO activities were also detectable in all liquid cultures with values up to ~10 U L^-1^ (S12 Fig). For the sake of completeness, three genes of intracellular class I peroxidases–two ascorbic acid peroxidases (APXs) and one cyctochrome c peroxidase (CcP)–were found in the *C*. *purpureum* genome as well (S9 Fig). The expression of CcP (*g2498*) was confirmed for BW cultures on the protein level (NSAF <0.01%).

A remarkable finding has been the high number of Lac genes (45, including 38 full-length sequences) predicted in the genome of *C*. *purpureum*. Phylogenetic analysis of the whole MCO family (including Lac, ferroxidase and pigment oxidase) revealed 40 full-length sequences and 14 partial sequences, which frequently were split on the end of contigs, i.e. showing only the N- or C terminus in an alignment (S13 Fig). Considering the 40 full-length genes, 38 can be defined as Lac *sensu stricto*. Highest identity between two full-length Lacs was 97% (S14 Fig) and more than half of all Lacs were expressed (in total 25, S13 Fig) with NSAF >0.01% in liquid and solid state cultures (23 and four proteins, respectively; Figs 2 and 3). Moreover, moderate Lac activities were detected during cultivations (up to ~10 U L^-1^; S11 and S12 Figs). The secretion of some of the Lacs was obviously stimulated in the presence of phenol-rich olive mill residues (e.g. *g1744* in SSF and *g9564*, *g6664*, *g5639*, *g1744* in KM with DOR and birch wood supplemented ASKM and BSKM, respectively). One MCO sequence turned out to belong to a Fet3 ferroxidase cluster (FeOX; *g718*) and another one to a fungal pigment MCO cluster (*g10148*; S13 Fig).

Further CAZy AA representatives (AA3-9) predicted in the genome rank among enzymes supporting peroxide-dependent biocatalysts acting on lignin and aromatics (i.e. oxidases that supply class II POD, UPO and DyP) or interacting with carbohydrates (GMC, GLX, LPMO); some of them occur with high relative abundance (4 to 36 genes; Table 3 & S4 Fig). The number of predicted proteins producing hydrogen peroxide (AA3 & AA5) was 48. Among this enzyme group, the only available CDH gene (AA3_1; *g6352*), encoding an enzyme involved in Fenton-based chemistry and LPMO activation, was expressed by *C*. *purpureum* in all culture media tested (Figs 2 and 3). Interestingly, the respective CDH gene product was in the solid-state cultures one of the most abundant proteins (NSAF ~0.9%) among the lignocellulose-relevant enzymes (Fig 2). From the AA3_2 group (GMCs), 24 genes encoding for glucose and aryl alcohol oxidases were predicted and the fungus expressed up to six of them in all media tested (Figs 2 and 3). In KM, the addition of DOR or birch wood (ASKM and BSKM) led to a higher relative abundance of some of the AAOs (e.g. *g12545*, *g8332*, *g8331*), which were also present in the soybean medium with and without additives. Furthermore, ten sequences of alcohol oxidases (AA3_3) and one pyranose oxidase gene (AA3_4) were identified in the genome (S4 Fig). Several genes of the former groups (e.g. *g12139*) were expressed both in SSF and SF. There is indication that the AA3_3 gene *g12139* could be constitutive, since it showed also high relative abundance in KM, in which eligible inducing compounds (e.g. aromatics from wood or DOR) were not present (Fig 3). The number of genes encoding for copper radical oxidases (CRO and GLX, AA5) was twelve, and some of them were expressed in SSF and SF with NSAF >0.01% (e.g. *g9579*, *g9433*, *g9062*, *g2075*). Eventually, four genes encoding for benzoquinone reductase (AA6) were found in the genome indicating that the enzymatic basis of Fenton-based attack on lignocellulose is realized in *C*. *purpureum*.

#### Taxonomy

**Fig 4:**
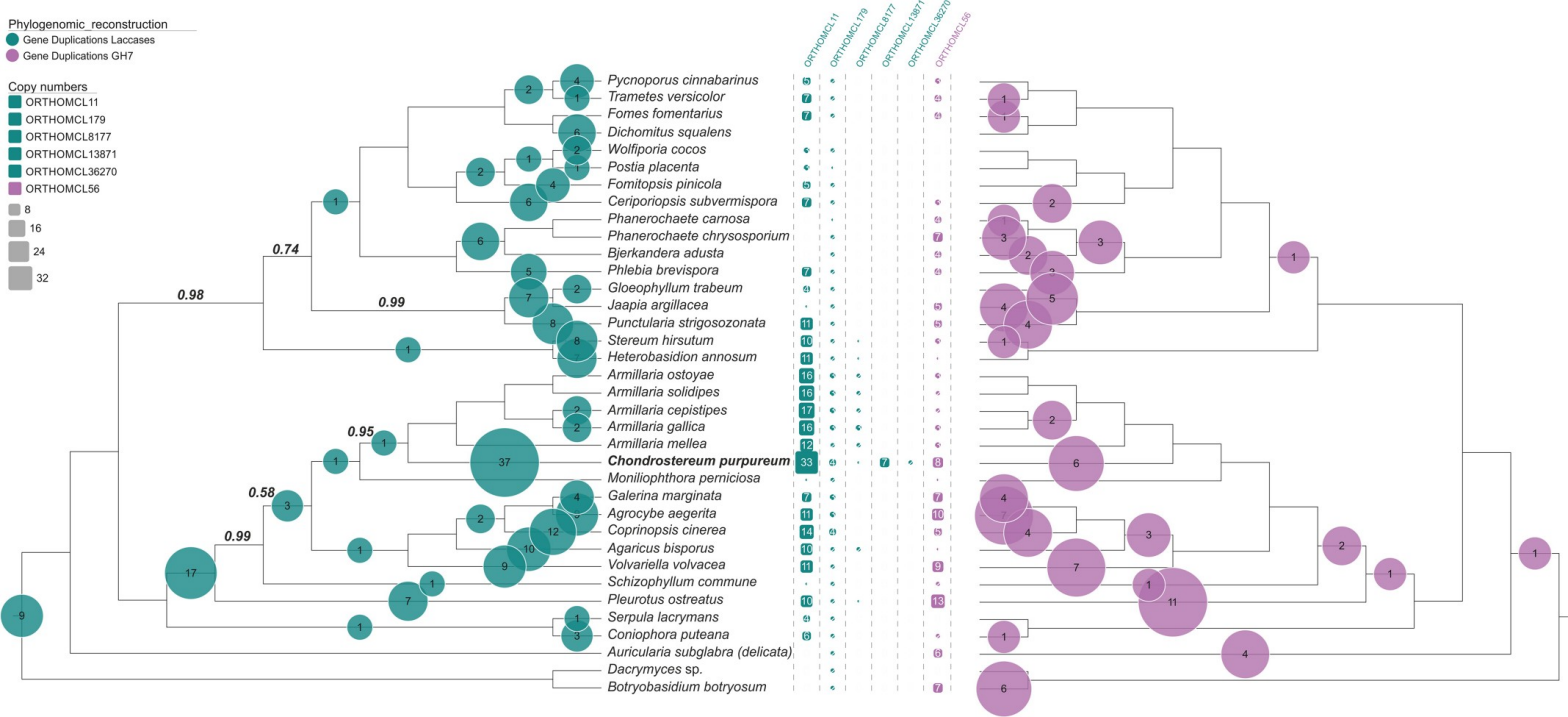
Genome-level species tree of 36 basidiomycete fungi inferred using ASTRAL [66]. All branches had posterior probabilities of 1, except where indicated otherwise above branches. Bubble plots show the numbers of inferred duplications across all orthogroups encoding Lacs (green, left) or GH7 cellobiohydrolases (purple, right). The middle section indicates the number of orthogroups housing genes in each of the respective classes and their copy number in each species.

A phylogenetic analysis that comprised of 609 single copy genes from 36 basidiomycete genomes placed *C*. *purpureum* in a clade close to *Moniliophthora perniciosa* (Marasmiaceae) and four *Armillaria* species (Physalacriaceae), which all represent distinct pathogens within the Agaricales (Fig 4). Complementary approaches using either ML reconstruction or a summary method based on the multispecies coalescent resulted in nearly identical, well resolved trees (Fig 4 and S15 Fig), with the exception of the branching order of *C*. *purpureum* and *M*. *perniciosa*. Currently only few genomes of the so called ‘marasmioid clade’ [19] are known and larger numbers of taxa in this clade will be required for accurate resolution of the relationship between *C*. *purpureum* and *M*. *perniciosa*. In analyses with a more dense taxon sampling but less phylogenetically relevant genes (two to six genes), a possible phylogenetic position to the Cyphellaceae was shown [19, 78]; the genes found in the assembled genome were identical to the previously analyzed *C*. *purpureum* genes in the study of Matheny and coworkers.

#### Gene content evolution

Inference of gene duplication (λ) and loss (μ) rates yielded global estimates of 9.27e-4 and 2.59e-3 events per unit branch length, respectively. A total of 25 orthogroups showed significant deviation from these genomic background rates on the *C*. *purpureum* branch (P <0.01), and in any case, this entailed expansions rather than contractions (Table 4). Approximately two thirds of significantly expanded families were attributable to transposable elements based on annotation with PFAM domains or BLAST searches. The most pronounced expansion among structural genes was ORTHOMCL11, encoding a family of Lac, in the case of which we inferred a total of 18 lineage-specific duplications using the model-based approach. Significantly expanded families also included a putative non-ribosomal peptide synthase, two families of proteases and a family of cytochrome P450s.

**Table 4:** Significantly expanded orthogroups in *C*. *purpureum*.

OrthoGroup	PFAM domains	Putative Function
ORTHOMCL2869[+4]	Plavaka (PF18759)	Likely transposable element
ORTHOMCL7313[+2]	None (RT based on BLAST)	Likely transposable element
ORTHOMCL383[+20]	None (Gag-Pol based on BLAST)	Likely transposable element
ORTHOMCL194[+21]	KDZ (PF18758)	Likely transposable element
ORTHOMCL305[+16]	CxC2 (PF18803)	Likely transposable element
ORTHOMCL7306[+15]	Transposase_21 (PF02992)	Likely transposable element
ORTHOMCL7397[+2]	None	Hypothetical protein
ORTHOMCL477[+16]	RVT_1 (PF00078)	Likely transposable element
ORTHOMCL480[+2]	KDZ (PF18758)	Likely transposable element
ORTHOMCL7175[+2]	None (FAR1 based on BLAST)	Likely transposable element
ORTHOMCL3789[+21]	None	-
ORTHOMCL88[+3]	RVT_1 (PF00078)	Likely transposable element
ORTHOMCL7296[+2]	None	-
ORTHOMCL404[+5]	PIF1 (PF05970)	Helicase—possibly TE
ORTHOMCL601[+2]	DUF4470 (PF14737)	-
ORTHOMCL330[+7]	Retrotrans gag (PF03732)	Likely transposable element
ORTHOMCL75[+4]	AMP-binding (PF00501)	Non-ribosomal peptide synthetase (NRPS)
ORTHOMCL812[+3]	None	-
ORTHOMCL526[+5]	None (RT from BLAST)	Likely transposable element
ORTHOMCL727[+3]	RVT_2 (PF07727)	Likely transposable element
ORTHOMCL80[+6]	Helitron-like N (PF14214)	Likely transposable element
ORTHOMCL86[+3]	Peptidase_M36 (PF02128)	Fungalysin metalloprotease
ORTHOMCL192[+5]	Peptidase_S8 (PF00082)	Subtilisin like protease
ORTHOMCL11[+18]	Cu-oxidase (PF00394)	Laccase
ORTHOMCL43[+4]	P450 (PF00067)	Cytochrome P450

Since both Lac and GH7 cellobiohydrolase were found in unusually high numbers in the *C*. *purpureum* genome, we also included a focused phylogenetic analysis of orthogroups encoding genes in these families (Fig 4). Among annotated Lac, 39 were found spread across five orthogroups, ORTHOMCL11 being the largest (Fig 4 and S1 Fig). Thirteen Lacs did not cluster into orthogroups, of which ten constituted partial gene models, suggesting that FastOrtho clustering mitigates the problem of fragmentation for downstream evolutionary analyses. All annotated GH7s were found in ORTHOMCL56 (Fig 4). Phylogenetic analyses confirmed model-based inference, showing an exceptionally large lineage-specific expansion of Lac in *C*. *purpureum* mainly driven by ORTHOMCL11. Similarly, five of the eight GH7 copies likely arose on the *C*. *purpureum* branch (based on six duplications and one loss; Fig 4). Patterns for GH6 were complex, since members of this family were spread among two different orthogroups, with two out of five copies constituting singletons (S16 Fig).

**Fig 5:**
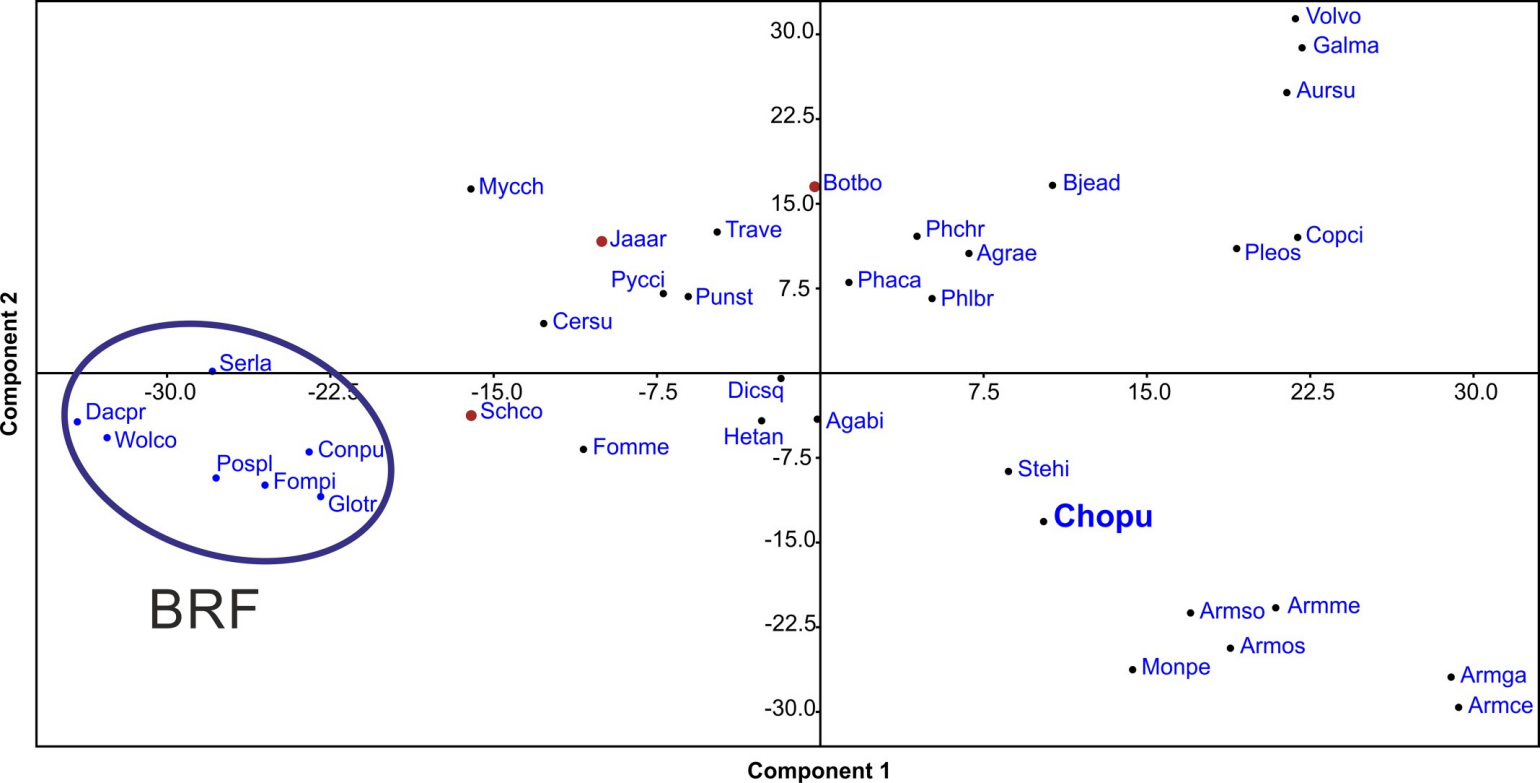
Principal component analysis of main CAZy lignocellulolytic gene content in the analysed genomes (published data from JGI & NCBI). A variance-covariance matrix of the values in (S4 Fig) were used. Agabi, *Agaricus bisporus*; Agrae, *Agrocybe aegerita* [60]; Armce, *Armillaria cepistipes*; Armga, *Armillaria gallica*; Armme, *Armillaria mellea*; Armso, *Armillaria solidipes*, Armos, *Armillaria ostoyae*; Aursu, *Auricularia subglabra*; Bjead, *Bjerkandera adusta*; Botbo, *Botryobasidium botryosum*; Cersu, *Ceriporiopsis subvermispora*; Chopu, *Chondrostereum purpureum*; Conpu, *Coniophora puteana*; Copci, *Coprinopsis cinereus*; Dacsp, *Dacryopinax sp*.; Dicsq, *Dichomitus squalens*; Fomme, *Fomitiporia mediterranea*; Fompi, *Fomitopsis pinicola*; Galma, *Galerina marginata*; Glotr, *Gloeophyllum trabeum*; Hetan, *Heterobasidion annosum*; Jaaar, *Jaapia argillacea*; Mycch, *Mycena chlorophos* [79]; Monpe, *Moniliophthora perniciosa*; Phaca, *Phanerochaete carnosa*; Phchr, *Phanerochaete chrysosporium*; Phlbr, *Phlebia brevispora*; Pleos, *Pleurotus ostreatus*; Pospl, *Postia placenta*; Punst, *Punctularia strigosozonata*; Pycci, *Pycnoporus cinnabarinus*; Schco, *Schizophyllum commune*; Serla, *Serpula lacrymans*; Stehi, *Stereum hirsutum*; Trave, *Trametes versicolor*; Volvo, *Volvariella volvacea* and Wolco, *Wolfiporia cocos*.

To classify the position of *C*. *purpureum* within genome-sequenced wood decomposing fungi, a principal component analysis was performed using the core lignocellulolytic genes (Fig 5). The axis 1 explained 44.3% of the variation and axis 2 explained 32%. Brown-rot fungi formed a distinct group, whereas white-rot and the so called ‘white-rot-like’ species (*Botryobasidium botryosum*, *Jaapia argillacea* and *S*. *commune*; all containing no class II peroxidase genes) formed a far more variable group (Fig 5). The lignocellulolytic gene content placed *C*. *purpureum* close to *Stereum hirsutum*, *M*. *perniciosa* and three *Armillaria* species. Conspicuous differences in the gene repertoire among the ecological classifications ‘white-rot’ (without the pathogenic species), ‘white-rot-like’, ‘pathogenic white-rot’ (*Armillaria* species, *M*. *perniciosa* and *C*. *purpureum*) and ‘brown-rot’ were found (S4 Fig). For example, class II peroxidases (AA2) occurred in average with seven genes in pathogenic WRF, twelve in the remaining WRF, and none in WRF-like and BRF. Class AA3_2 occurred on average with 52 genes in pathogenic WRF, 27 in the remaining WRF, 18 in WRF-like and 13 in BRF. Class AA1_1, Lac, occurred on average with 27 genes in pathogenic WRF, nine in the remaining WRF, one in WRF-like and four in BRF (S4 Fig).

## Discussion

### Phylogenetic position of *C*. *purpureum*

*C*. *purpureum* has been placed in the Cyphellaceae within the so called ‘marasmioid clade’ by Matheny and coworkers [19], and the current databases (MycoBank, Index Fungorum) follow this taxonomic affiliation. A follow-up study using a six gene approach could not further resolve the positioning of *C*. *purpureum* [78]. In our approach using more than 600 single copy genes, it has not been expected to reach further resolution, since only a ‘handful’ of genomes is currently being available from the ‘marasmioid clade’. Nevertheless, the identified genes of *C*. *purpureum* perfectly match with those of both studies of Matheny et al. [19, 78], and hence support its current phylogenetic position. Thus, the *C*. *purpureum* genome presented here will contribute to future studies dealing with the phylogeny of the marasmioid clade by using comparative genomics.

### Repertoire of genes and secreted proteins related to lignocellulose degradation in comparison to other wood-rot fungi

In addition to the *C*. *purpureum* genome, we provide insights into one of the few existing secretomes of fungi that can switch between phytopathogenic and saprotrophic life-styles. To obtain a broad proteomic data set, the fungus was cultured in liquid synthetic and plant-based complex media as well as in solid media containing wood and/or olive-wastes to follow enzyme secretion under different degradation and detoxification strategies.

The size of the *C*. *purpureum* genome (46.5 Mbp) is within the typical range of other fungi’s genomes, especially basidiomycetes (40–50 Mbp; [80]). On the other hand, the fungus secreted a high number of proteins (981 to 1,151 in dependence of the culture medium) in comparison to other basidiomycetes [27]. For example, following numbers of secreted proteins were reported for some other basidiomycetes: 243 for *Ceriporiopsis subvermispora* [81], 293 for *A*. *mellea* [33], 356 and 413 for *P*. *chrysosporium* and *P*. *placenta*, respectively [82] as well as 508 for *P*. *ostreatus* [27] when growing on lignocellulose-containing or synthetic media. An equally large number of secreted proteins (with almost 800) was reported for the secretome of *P*. *chrysosporium* when growing on the wood of three different poplar genotypes [83]. However, it should be taken into consideration that steady improvements in mass spectrometry may have influenced these data sets [84].

The fungus *C*. *purpureum* possesses in the genome and secretes diverse hydrolases and oxidoreductases that are involved in the degradation and modification of lignocelluloses and are typical for saprotrophic fungi. *C*. *purpureum* pertains to the ecological group of ‘pathogenic white-rot’ fungi like *Armillaria* spp. or *M*. *perniciosa* as indicated by a PCA considering core lignocellulolytic genes. *Sensu lato*, *C*. *purpureum* belongs to the WRF ecotype, incorporating key enzymes of lignin attack like manganese peroxidase.

#### Glycoside hydrolases

Although cellobiohydrolases of the families GH6 and GH7 are known to correlate with white-rot, the number of six genes belonging to family GH6 in the *C*. *purpureum* genome is one of the highest reported so far, considering all available data on WRF and BRF (<5 genes). The number of GH7 genes was nine and thus ranges in the middle of reported numbers for other WRF (e.g. one and 16 genes for *Heterobasidion annosum* and *P*. *ostreatus*, respectively). In this context, the fungus houses the highest number of GH7s in its clade and six lineage-specific duplications in this family (Fig 4). This is contrary to BRF, in which genes encoding these enzymes are rarely present and only few of them have one or two respective enzymes (GH6 or GH7) available. The presence of cellulases (families GH6 and GH7) in the secretome of *C*. *purpureum* is also a characteristic that it shares with other WRF (e.g. *P*. *chrysosporium*, *C*. *subvermispora*; [81, 83]), for which these enzymes were shown to be major proteins secreted in wood-containing media. These enzymes act on ‘bulky’ polysaccharide backbones and are seemingly up-regulated in *C*. *purpureum* when it grows in soybean-based medium for more than two weeks.

It has been accepted that seven CAZy families preferably target complex hemicelluloses (e.g. GH10, GH11, GH30), and eleven families the even more heterogeneous pectins (e.g. GH43, GH28) [21, 85]. Most of these enzymes were found to be present in the *C*. *purpureum* genome except families GH11 and GH26. Highly abundant GHs found in the *C*. *purpureum* secretomes of liquid cultures containing wood or ADOR belong to the families GH3 (*g3528*), GH5 (*g6251*, *g7580*) and GH10 (*g13143*). These proteins were also reported to be over-produced in other WRF in the presence of lignocellulosic substrates [27]. GH5 represents a large protein family that contains a range of enzymes acting on β-linked oligo- and polysaccharides [64], and 17 out of 25 genes present in the genome were expressed by *C*. *purpureum*, predominantly in soybean-based medium.

Pectinolytic hydrolases expressed by *C*. *purpureum* in SF (e.g. GH28; CE4, 8, 9 and 15) play probably a role during the invasive step of fungal plant pathogens as proposed for typical representatives such as *Botrytis cinerea* and *Fusarium oxysporum* [14, 21, 86]. The GH28 family comprises endo- and exo-[rhamno]galacturonases, essential for pectinolysis by both parasitic and saprotrophic fungi [87]. Genes encoding such enzymes that disintegrate the middle lamellae of plant cell walls, are highly abundant in facultative parasites (i.e. WRF that are both necrotrophic and biotrophic such as the tree pathogen *A*. *mellea* that contains 17 respective genes in its genome) and are just moderately represented in exclusively saprotrophic fungi (e.g. dead-wood dwellers such as *C*. *subvermispora* with six and *P*. *chrysosporium* with four respective genes; [33, 87]).

The expressed CE proteins of *C*. *purpureum*–found in the secretome of *A*. *mellea* as well–are also involved in the degradation of pectins, e.g. by members of the CE8 family, which cleave methyl esters. CE4 is a chitin deacetylase and acts on the acetyl group of *N*-acetyl glucosamine [33]. It may cooperate with fungal chitinase, e.g. GH18 of *C*. *purpureum*, to deacetylate chitin to chitosan oligomers that do not longer elicit a plant defence response, and thus enabling the fungus to invade living plants/trees without any appreciable resistance [88]. The high abundance of pectinolytic enzymes and the presence of chitin-degrading enzymes in the genome and secretome of *C*. *purpureum* implies that they are part of its pathogenic system causing the silver-leaf disease ([89, 90], originally described to be caused by *‘Stereum’ purpureum* [91]).

#### Oxidoreductases

The number of LPMO genes (31) is in the upper range of that of other WRF like *B*. *adusta* (Meruliaceae, 28 genes; [44]) or *P*. *ostreatus* (Pleurotaceae, 29 genes; [21]) and leaf-litter decomposers such as *Coprinopsis cinerea* (Psathyrellaceae, 34 genes; [56]). Even in ‘atypical’ wood-rot fungi, high numbers of LPMO genes can be found (e.g. 32 genes in *B*. *botryosum*), while in BRF, the number of LPMO genes generally goes below ten [21]. LPMOs are thought to be involved in the oxidative cleavage of recalcitrant wood polysaccharides, particularly in that of crystalline cellulose [92] but may have also completely different roles, for example, during chitin development in ancient arthropods [93]. The expression levels of LPMOs reported for wood-degrading fungi differ considerably. Thus, during growth on woody substrates, almost half of the existing 15 genes were expressed in *P*. *chrysosporium* [94]; *Phlebia gigantea* expressed six out of 15 genes [95], *C*. *subvermispora* five out of nine genes [81] and *P*. *radiata* seven out of twelve [96]. On the other hand, only low or even no expression of LPMOs was observed for *Wolfiporia coccos* [97] and *P*. *ostreatus* [27] under comparable conditions. LPMOs are capable of enhancing oxidative attack on crystalline cellulose by cooperating with CDH [98]. The high abundance of LPMO in SF of *C*. *purpureum* points to a certain LPMO-stimulating effect by plant secondary ingredients and hemicellulose derivatives [39, 40, 99]. Furthermore, it could be an indication for their joint action with CDH that functions as an activating electron donor for LPMO and thereby improving the incipient degradation of crystalline cellulose [98]. Moreover, CDH has been proposed to play a role in the generation of hydroxyl radicals (HO•) *via* Fenton chemistry and thereby may also contribute to the degradation of cellulose as well as the modification of lignin [100]. CDH is uniformly present as a single gene copy in all WRF but absent in the majority of BRF [21, 22, 100]. High expression of CDH in relation to lignocellulose degradation (e.g. of aspen wood or wheat straw) was reported for *P*. *chrysosporium* [94], *I*. *lacteus* [101], *P*. *gigantea* [95], *C*. *subvermispora* [81] and *P*. *radiata* [96]. In contrast to these findings, CDH was not detectable in secretomes of *P*. *ostreatus* grown on poplar wood or wheat straw.

The presence of high-redox potential class II PODs (i.e. MnPs) in the genome of *C*. *purpureum* fulfills a well-known characteristic of all WRF and is a prerequisite for the efficient degradation of lignin. BRF, on the other hand, are completely lacking these biocatalysts similar as some ‘uncategorized’ (atypical or ‘white-rot-like’) wood-rot fungi [21]. Ligninolytic PODs can occur in various combinations of MnP, VP and LiP (and there are several subtypes of MnPs with somewhat different substrate spectra; [58, 102]. The number of MnP genes in white-rot fungi ranges from two in *A*. *bisporus* to 13 and 16 in *C*. *subvermispora* and *Fomitoporia mediteranea*, respectively [21, 81]. *C*. *purpureum* with its two MnP encoding genes rather groups in the WRF clade with few MnP genes in this classification system. As an agaric WRF, *C*. *purpureum* is lacking LiP genes; the occurrence of LiP is seemingly restricted to the evolutionary old order of Polyporales, for example, to species like *T*. *versicolor*, *P*. *chrysosporium*, *P*. *radiata* or *B*. *adusta* [58].

Among the two ‘short-type’ MnPs of *C*. *purpureum*, only one was expressed and only in liquid culture. Similarly, the necrotrophic WRF *H*. *irregulare* possesses six short MnPs out of seven class II PODs but none of them showed strong expression when the fungus grew on spruce heartwood [32]. For *P*. *gigantea*, a similar observation was made; from seven genes encoding MnPs, the expression level in the corresponding secretome was rather low during growth on acetone-extracted and non-extracted pine wood [95]. A possible explanation for the low expression level of MnP genes in *C*. *purpureum* could be related to the fungus’ affiliation to the group of ‘agaric’ WRF, which seemingly possess a simpler ligninolytic enzyme system compared to ‘polyporous’ WRF. *Volvariella volvacea* (Rice straw mushroom) with its two MnPs can serve as a typical example of such fungi [57]. Possibly, lignin degradation by fungi with small numbers of class II peroxidases may be supported by other oxidative enzymes and/or mechanisms. Furthermore, MnPs are known to be secreted during early stages of fungal lignocellulose degradation (e.g. second to fourth week [103]) so that they may have already disappeared at the time point when the *C*. *purpureum* secretome was analyzed (after six weeks).

Interestingly also HTPs, with UPOs as their most prominent representatives, were found to be present in the *C*. *purpureum* genome. In general, these enzymes are widespread among the whole fungal kingdom including all phyla of true fungi (Eumycota) and a few ‘pseudofungal’ stramenopiles (‘Oomycota’) [77]. However, it is not much known on their physiological functions and on the natural substances that regulate their expression [104]. *C*. *purpureum* expressed five out of six UPOs, which all belonged to the phylogenetically older ‘short’ type genes. Because of their catalytic versatility, it is conceivable that UPOs may be involved in extracellular detoxification reactions, e.g. of low molecular weight compounds typically found in living plants, wood, and soil (secondary metabolites, tannins, phytoalexins, microbial toxins, xenobiotics, etc.). In addition to triggering UPO production by soybeans and their ingredients in different fungi (e.g. by soybean meal, [48]; soybean peptone, [105]; glycinin or conglycinin, [106]), recently published data indicate that DOR and its aqueous extract (ADOR) have stimulating effects on UPO secretion too [38, 107].

Two of six genes encoding for DyPs were expressed by *C*. *purpureum*, but both only in beech-wood supplemented soybean medium and in solid-state cultures; in the latter case, the corresponding NSAF (%) was higher in beech-wood (BW) than in respective solid cultures supplemented with DOR (BWD). It cannot be ruled out that both types of peroxide-using enzymes, UPOs and DyPs, are partially overlapping with class II PODs in their ability to oxidize lignin structures, and thus, they should be included in the pool of lignin-modifying enzymes [46–48, 108, 109].

Peroxide generating enzymes (e.g. AA3 and AA5) constitute another important component of the ligninolytic system of wood-rot fungi, since H_2_O_2_ is required as the oxidant (electron acceptor) in peroxidative (and peroxygenating) reactions. The high abundance of respective genes/proteins in *C*. *purpureum*‘s genome/secretome reflects this fact and indirectly points to the importance of peroxidase-based processes during wood decay [110, 111].

There are other oxidoreductases that are thought to be involved in lignin modification. The most prominent ones are Lacs being abundant in most WRF (though they are lacking in the model organisms *P*. *chrysosporium* and *B*. *adusta*; [5, 112]) and they also occur in some BRF [21]. Taking into account all information from fungal genomes, *C*. *purpureum* has, with 45 sequences in total, the largest number of Lac genes so far reported for a fungus. It exceeds the 23 to 25 Lac genes of the phylogenetically related, phytopathogenic *Armillaria* species, which were hitherto the ‘record holders’ in terms of the number of Lac genes [113, 114]. Birth-death model based analyses of gene content evolution pinpoint Lacs as some of the most rapidly evolving gene families in *C*. *purpureum*, showing significant lineage-specific expansion in this species (Table 4). This was confirmed by sequence-based analyses, in the course of which we inferred 37 lineage-specific duplications in five families (Fig 4). High numbers of Lac genes were also found in the genomes of the biotrophic (parasitic) fungi *H*. *annosum* (14 genes, [21]), *H*. *irregulare* (18 genes, [115]) and *M*. *perniciosa* (24 genes, [55]). Remarkably, the high abundance of Lac genes was adequately reflected by the expression patterns of *C*. *purpureum* cultures comprising 25 Lac proteins in total. This has led to the assumption that Lacs are not only involved in fruiting body development [116] and mycelial growth [117] but may be also active in both parasitic and saprotrophic life styles of the fungus (e.g. during plant tissue invasion or cell wall modification; [55]). Such role of Lacs in virulence (e.g. by detoxifying phenolic compounds during host-defense) has already been postulated for *A*. *mellea* and *H*. *annosum* [32, 33, 115], and also for phytopathogenic Ascomycota like *Colletotrichum* spp. and *Sclerotinia* spp. [118, 119] [94, 95] and even for human pathogens like *Talaromyces marneffei* [120].

A further genomic feature that may be associated with *C*. *purpureum*’s phytopathogenic life style is the significant expansion of non-ribosomal peptide synthetases (NRPSs), proteases and cytochrome P450 (Table 4), all of which were also found to be expanded in pathogenic *Armillaria* spp. highlighting the dual ecology of this genus [26]. The whole repertoire of genes–with expansions, for example, in Lacs, GH7 and proteases combined with relative high abundances of pathogenicity and lignocellulose-related proteins (e.g. Lacs, GH7, AA3_3) in different culture media–is, from our point of view, indicative for the switching lifestyle of *C*. *purpureum* and suggests its adaptability to changing environmental conditions. It confirms the affiliation of *C*. *purpureum* to the group of facultative parasitic agarics among the WRF (along with *Armillaria* spp. and *M*. *perniciosa*), which are notorious for their changing lifestyles.

## Conclusion

*C*. *purpureum* is a member of the order Agaricales, family Cyphellaceae within the current taxonomic classification. According to its genome, the fungus has a diverse genetic repertoire of heme peroxidases (including MnPs, HTPs/UPOs and DyPs), LPMOs, Lacs as well as of H_2_O_2_-generating oxidases. The presence of a single CDH, CBMs as well as of diverse cellulolytic and hemicellulolytic hydrolases perfectly matches the eco-physiological classification of *C*. *purpureum* as a WRF. Secretomic analyses revealed that the fungus realizes production and secretion of all these enzymes degrading and modifying lignocelluloses during growth in different complex solid and liquid media. Some of these media facilitated higher protein abundances in the presence of special medium components such as soybean meal (mainly with respect to GH6, seven LPMOs and one UPO, *g5554*), milled wood or phenol-rich agro-wastes (mainly for CBMs, PLs and LPMOs). These findings, along with the close relation of *C*. *purpureum* to other wood-rot agarics with both phytopathogenic and saprotrophic life styles, implies a particular role of these enzymes in transformation and detoxification of plant secondary metabolites, in a way of self-protection and manipulation of the environment in order to overcome plant resistance mechanisms. At the same time, they are responsible for the disintegration of biopolymers in the fungus’ micro-environment and thus contribute to its acquisition of nutrients. Not least, in that context, *C*. *purpureum* seems to be a suitable candidate for further biotechnological studies regarding the disintegration and modification of recalcitrant materials.

## Supporting information

S1 Fig Remapping of raw reads to the assembly. Examples are given for few full length laccase, GH6 and GH7 genes. Coverage is given on the top in blue, tracks indicate intro-exon and CDS structure and the red arrow indicates the track with the non-synonymous SNP positions.
(TIF)

S2 Fig Gene sequences of *C. purpureum* classified according Gene Ontology domains: (left) biological process, (middle) molecular function and (right) cellular component. (TIF)

S3 Fig Assignment of gene sequences of *C. purpureum* to main enzyme classes according to the EC nomenclature. (TIFF)

S4 Fig Lignocellulose-degrading enzymes in different basidiomycetous wood-decay fungi (modified according to Riley et al., 2014; published data from JGI & NCBI), yellow–WRF, grey–BRF, blue–unresolved wood-rot fungi. Agabi, *Agaricus bisporus*; Agrae, *Agrocybe aegerita* (Gupta et al. 2018); Armce, *Armillaria cepistipes*; Armga, *Armillaria gallica*; Armme, *Armillaria mellea*; Armso, *Armillaria solidipes*, Armos, *Armillaria ostoyae*; Aursu, *Auricularia subglabra*; Bjead, *Bjerkandera adusta*; Botbo, *Botryobasidium botryosum*; Cersu, *Ceriporiopsis subvermispora*; Chopu, *Chondrostereum purpureum*; Conpu, *Coniophora puteana*; Copci, *Coprinopsis cinereus*; Dacsp, *Dacryopinax sp*.; Dicsq, *Dichomitus squalens*; Fomme, *Fomitiporia mediterranea*; Fompi, *Fomitopsis pinicola*; Galma, *Galerina marginata*; Glotr, *Gloeophyllum trabeum*; Hetan, *Heterobasidion annosum*; Jaaar, *Jaapia argillacea*; Mycch, *Mycena chlorophos* (Tanaka et al. 2014); Monpe, *Moniliophthora perniciosa*; Phaca, *Phanerochaete carnosa*; Phchr, *Phanerochaete chrysosporium*; Phlbr, *Phlebia brevispora*; Pleos, *Pleurotus ostreatus*; Pospl, *Postia placenta*; Punst, *Punctularia strigosozonata*; Pycci, *Pycnoporus cinnabarinus*; Schco, *Schizophyllum commune*; Serla, *Serpula lacrymans*; Stehi, *Stereum hirsutum*; Trave, *Trametes versicolor*; Volvo, *Volvariella volvacea* and Wolco, *Wolfiporia cocos*; *generic peroxidase, ! probably a generic peroxidase.
(PDF)

S5 Fig Relative protein abundance (% NSAF) of enzymes produced by *C. purpureum* during solid-state fermentation (SSF). Beech-wood (BW, inner ring) and beech-wood plus DOR (BWD, outer ring). CAZy proteins are highlighted in bold letters. Organelle proteins include ribosomal, peroxisomal and vacuolar proteins without defined catalytic properties. Values are the mean of three replicates.
(TIF)

S6 Fig Relative protein abundance (% NSAF) of enzymes produced by *C. purpureum* during (submerged) liquid fermentation (SF). (A) KM (inner ring) ASKM, (middle ring) and BSKM (outer ring) and (B) SM (inner ring), ASSM (middle ring) and BSSM (outer ring). CAZy proteins are highlighted in bold letters. Organelle proteins include ribosomal, peroxisomal and vacuolar proteins without defined catalytic properties. Values are the mean of three replicates.
(TIF)

S7 Fig Heat map of the secreted CAZymes of *C. purpureum* that show significant differences during SF in KM, SM, ASKM, ASSM, BSKM and BSSM. Differences between treatments were corroborated with Hotelling’s T2 test. Abundance is demonstrated by the normalized spectral abundance factor (% NSAF). GHs are shown in the upper side while the rest of the CAZymes (including AAs) are given in the lower side.
(PDF)

S8 Fig Principal component analysis (PCA) bi-plot of the *C. purpureum* secretome from SSF cultures. (**left**; BW and BWD loadings are highlighted in red) and SF cultures (**right**; SM, ASSM, BSSM, KM, ASKM and BSKM loadings are highlighted in red) using NIPALS algorithms.
(PDF)

S9 Fig Neighbor-Joining phylogenetic tree of 54 class I and II peroxidase protein sequences (manganese, lignin, versatile and generic peroxidases (MnP, LiP, VP, GP), ascorbate peroxidases (APX) and cytochrome c peroxidases (CcP)). The sequences of *C*. *purpureum* are marked by a “g” and in bold. Numbers with asterisks indicate proteins detected in the secretomes; only complete sequences were considered. Sequences were aligned with Clustal W and Jukes-Cantor distance models were used.
(TIF)

S10 Fig Alignment of a versatile peroxidase of *Pleurotus eryngii* (2BOQ_A) with the class II peroxidases of *C. purpureum*. The three acidic amino acid residues typical for the manganese-binding sites of MnPs are indicated.
(TIF)

S11 Fig **Time course of extracellular oxidoreductase production by *C*. *purpureum* during solid-state fermentation (SSF) of cultures containing beech wood (BW, left) and beech wood supplemented with olive-mill residues ‘DOR’ (BWD, right)**; manganese-dependent peroxidase activities (MnP, squares), unspecific peroxygenase (UPO, circles) and laccase activities (Lac, triangles) and pH (dashed line).
(PDF)

S12 Fig **Time course of extracellular oxidoreductase production by *C*. *purpureum* during SF in cultures containing (a) Kirk medium (KM) and (b) soybean meal suspension (SM), (c) KM-ADOR (ASKM), (d) SM-ADOR (ASSM), (e) KM-birch wood (BSKM) and (f) SM-birch wood (BSSM)**; manganese-dependent peroxidase activities (MnP, squares), unspecific peroxygenase (UPO, circles) and laccase activities (Lac, triangles).
(PDF)

S13 Fig Neighbor-Joining phylogenetic tree of 67 multicopper oxidases (MCO) protein sequences (laccase (Lac), ferroxidase (FeOX), ascorbate oxidase (ASC). The sequences of *C*. *purpureum* (38 full length and 3 partial sequences (from C-terminal)) are marked by an “g” and in bold. Numbers with asterisks indicate that the proteins were found in the secretome. Sequences were aligned by Clustal W and Jukes-Cantor distance model were used.
(TIF)

S14 Fig Alignment of all full-length and partial MCO genes. The alignment was performed using ClustalW with parameters: BLOSUM cost matrix, gap opening cost = 10, gap extension cost = 0.1.
(TIF)

S15 Fig Maximum likelihood phylogeny. Maximum Likelihood phylogeny based on 609 genes across 36 genomes. Numbers indicate bootstrap support at each node.
(PDF)

S16 Fig Numbers of inferred duplications across all orthogroups encoding GH6 cellobiohydrolases. The middle section indicates the number of orthogroups housing genes in each of the respective classes and their copy number in each species. Agabi, *Agaricus bisporus*; Agrae, *Agrocybe aegerita*; Armce, *Armillaria cepistipes*; Armga, *Armillaria gallica*; Armme, *Armillaria mellea*; Armso, *Armillaria solidipes*, Armos, *Armillaria ostoyae*; Aursu, *Auricularia subglabra*; Bjead, *Bjerkandera adusta*; Botbo, *Botryobasidium botryosum*; Cersu, *Ceriporiopsis subvermispora*; Chopu, *Chondrostereum purpureum*; Conpu, *Coniophora puteana*; Copci, *Coprinopsis cinereus*; Dacsp, *Dacryopinax sp*.; Dicsq, *Dichomitus squalens*; Fomme, *Fomitiporia mediterranea*; Fompi, *Fomitopsis pinicola*; Galma, *Galerina marginata*; Glotr, *Gloeophyllum trabeum*; Hetan, *Heterobasidion annosum*; Jaaar, *Jaapia argillacea*; Mycch, *Mycena chlorophos*; Monpe, *Moniliophthora perniciosa*; Phaca, *Phanerochaete carnosa*; Phchr, *Phanerochaete chrysosporium*; Phlbr, *Phlebia brevispora*; Pleos, *Pleurotus ostreatus*; Pospl, *Postia placenta*; Punst, *Punctularia strigosozonata*; Pycci, *Pycnoporus cinnabarinus*; Schco, *Schizophyllum commune*; Serla, *Serpula lacrymans*; Stehi, *Stereum hirsutum*; Trave, *Trametes versicolor*; Volvo, *Volvariella volvacea* and Wolco, *Wolfiporia cocos*.
(PDF)
