# Supplementary figures and images for: Genome and secretome of Chondrostereum purpureum correspond to saprotrophic and phytopathogenic life styles
Source: PLoS One. 2019 Mar 1;14(3):e0212769. doi: 10.1371/journal.pone.0212769 (PMC6396904; doi:10.1371/journal.pone.0212769)

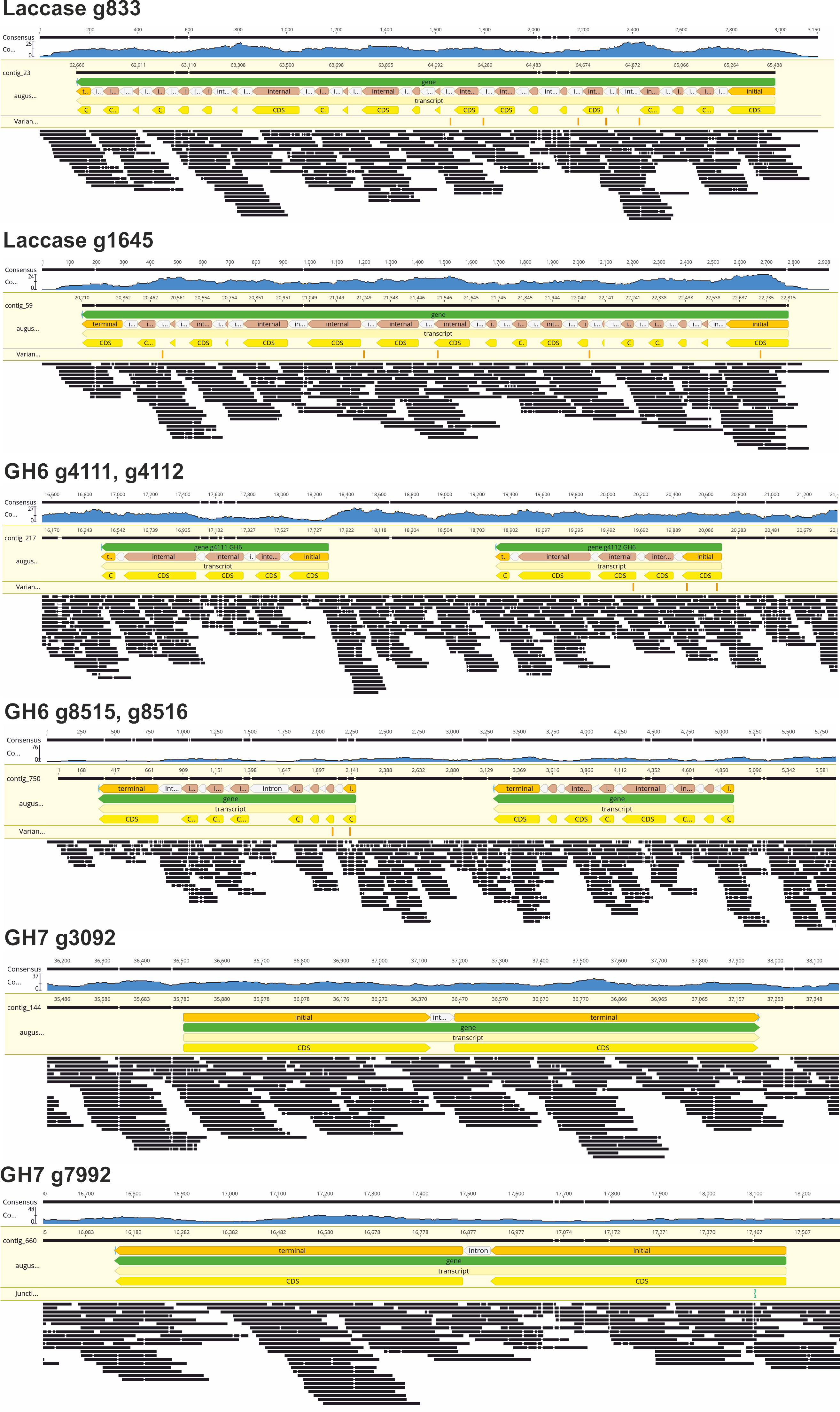

Supplement: S1 Fig — Examples are given for few full length laccase, GH6 and GH7 genes. Coverage is given on the top in blue, tracks indicate intro-exon and CDS structure and the red arrow indicates the track with the non-synonymous SNP positions. (TIF) [file pone.0212769.s001.tif]

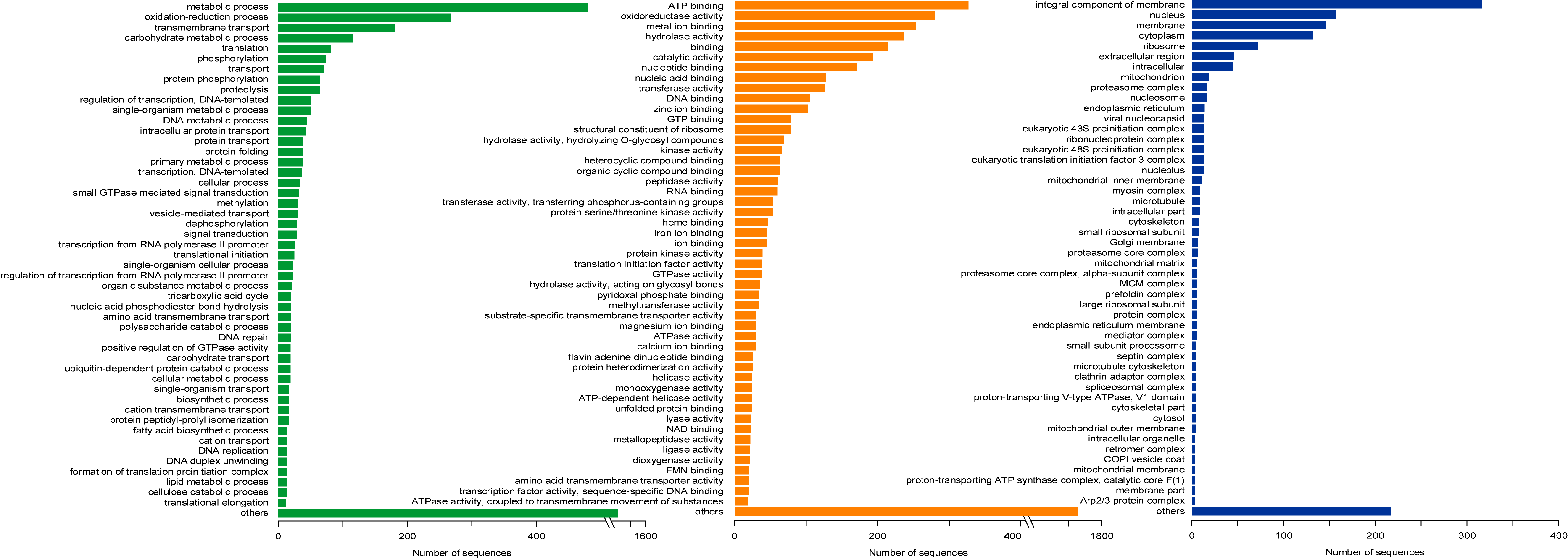

Supplement: S2 Fig — (TIF) [file pone.0212769.s002.tif]

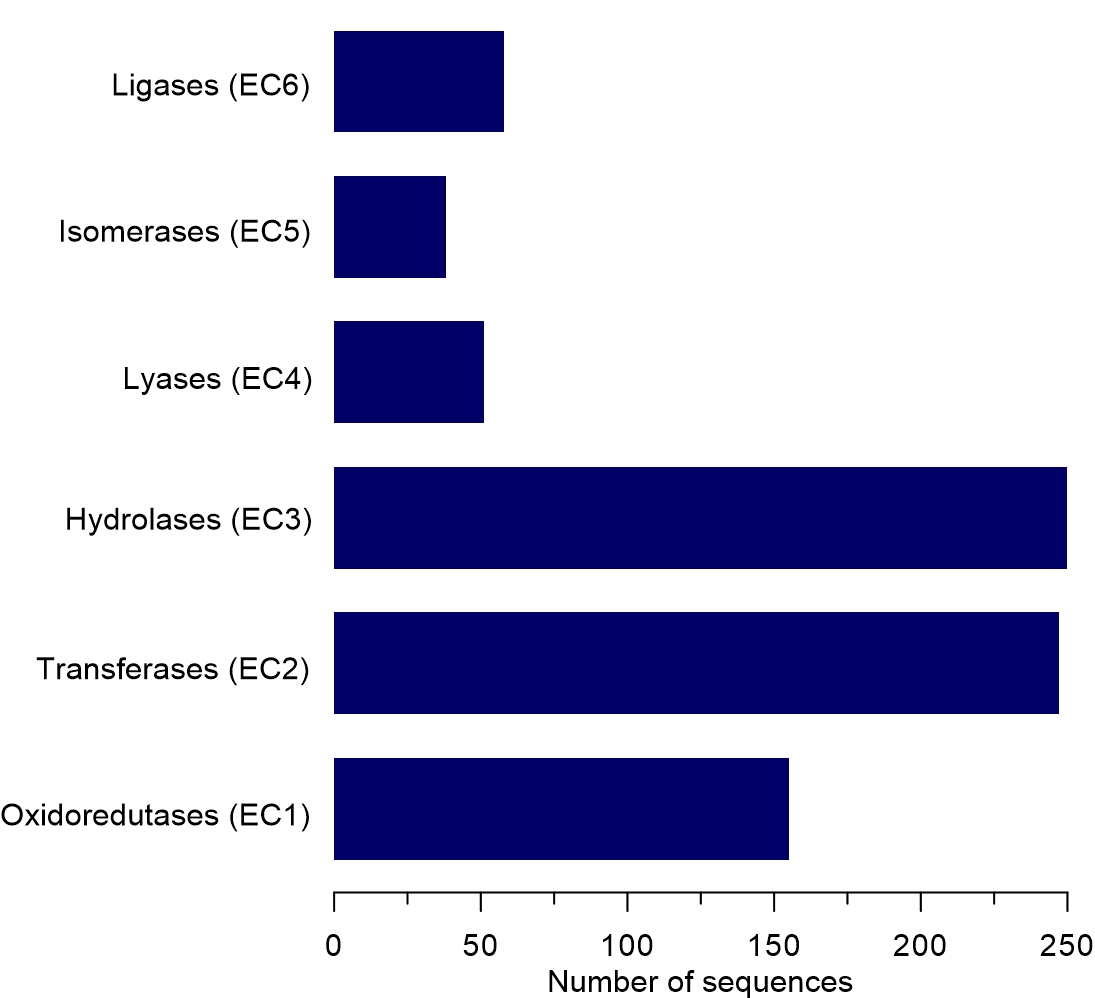

Supplement: S3 Fig — (TIFF) [file pone.0212769.s003.tiff]

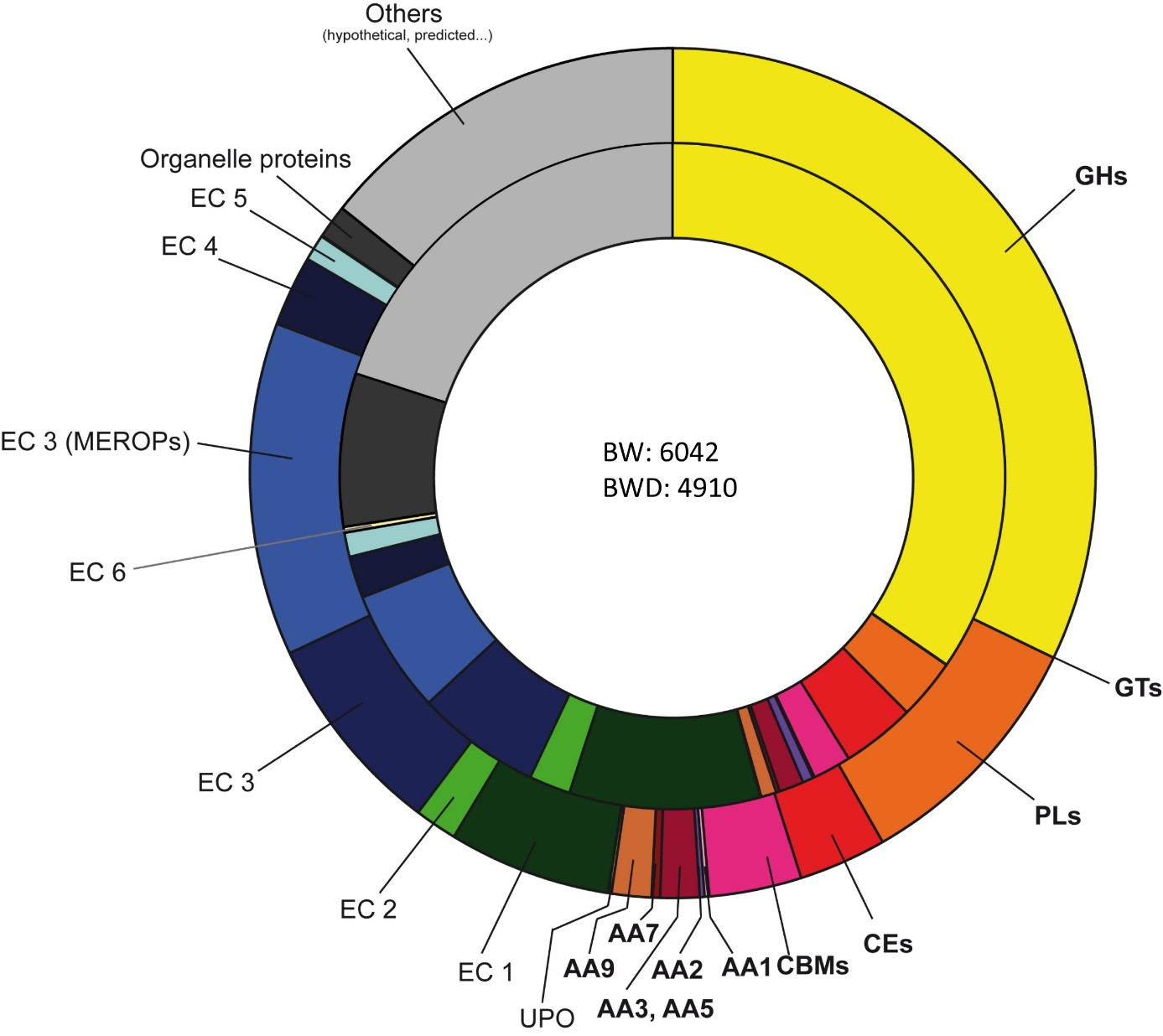

Supplement: S5 Fig — Beech-wood (BW, inner ring) and beech-wood plus DOR (BWD, outer ring). CAZy proteins are highlighted in bold letters. Organelle proteins include ribosomal, peroxisomal and vacuolar proteins without defined catalytic properties. Values are the mean of three replicates. (TIF) [file pone.0212769.s005.tif]

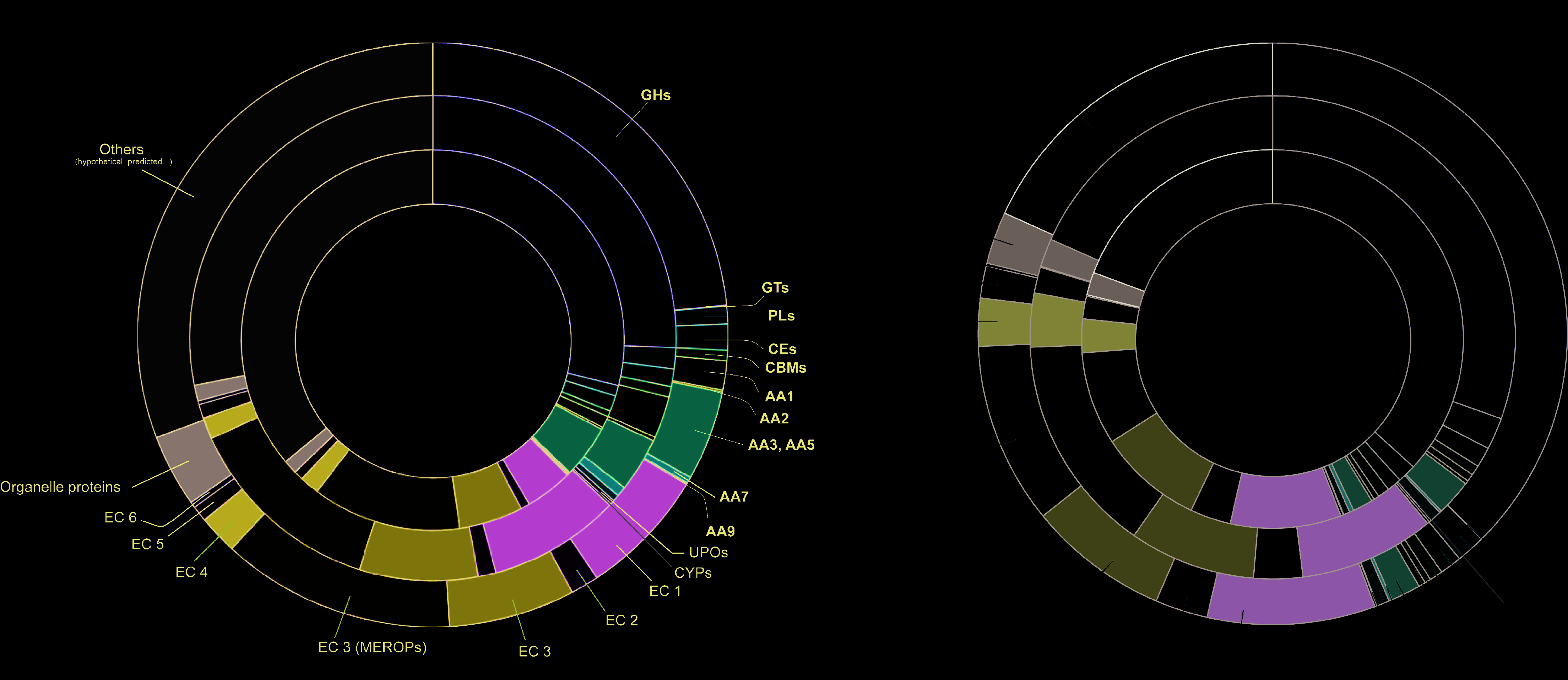

Supplement: S6 Fig — (A) KM (inner ring) ASKM, (middle ring) and BSKM (outer ring) and (B) SM (inner ring), ASSM (middle ring) and BSSM (outer ring). CAZy proteins are highlighted in bold letters. Organelle proteins include ribosomal, peroxisomal and vacuolar proteins without defined catalytic properties. Values are the mean of three replicates. (TIF) [file pone.0212769.s006.tif]

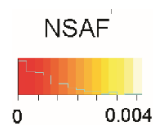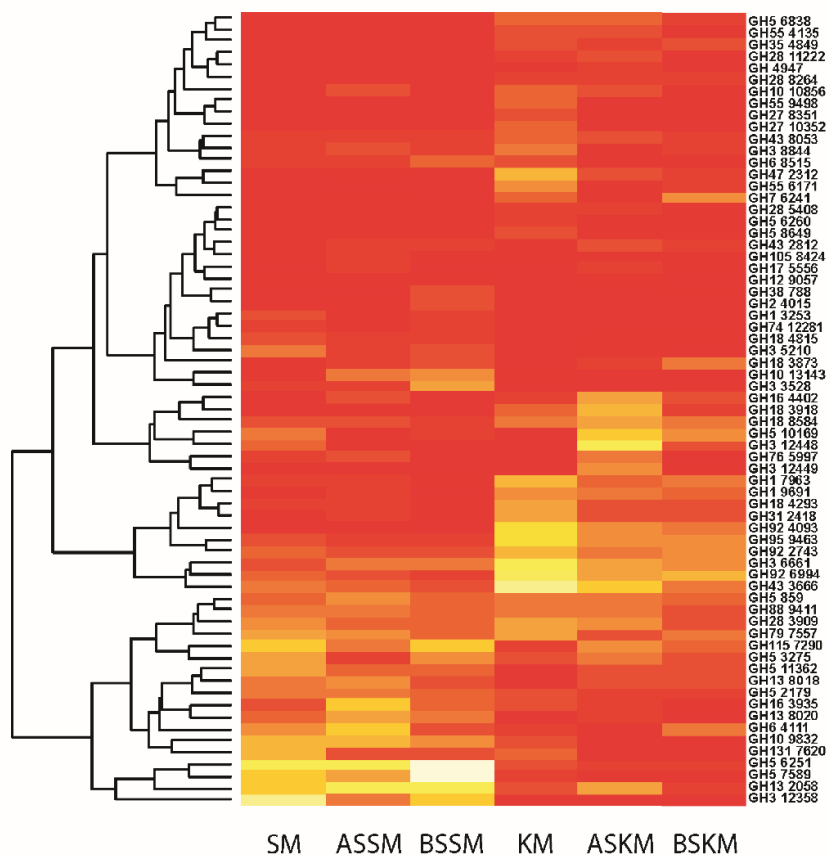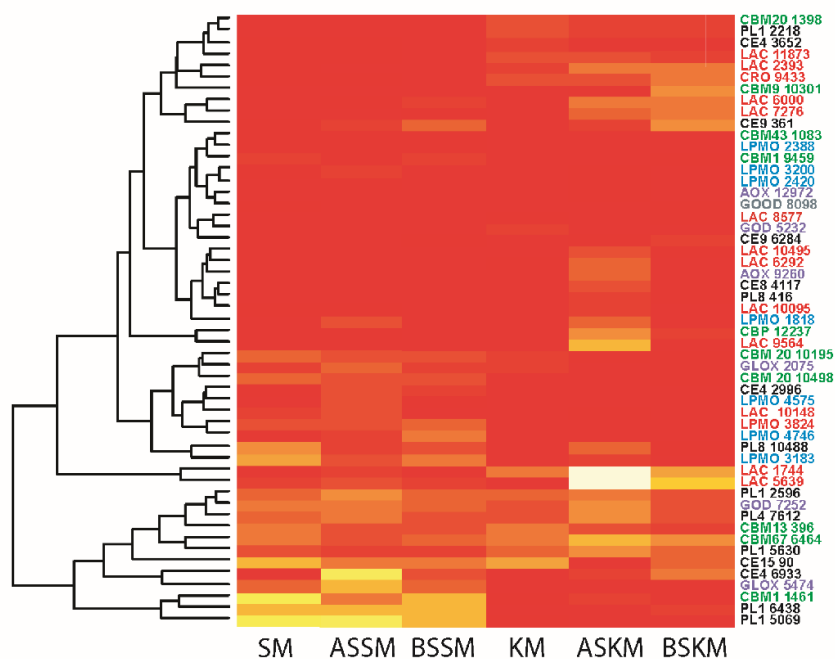

Supplement: S7 Fig — Differences between treatments were corroborated with Hotelling’s T2 test. Abundance is demonstrated by the normalized spectral abundance factor (% NSAF). GHs are shown in the upper side while the rest of the CAZymes (including AAs) are given in the lower side. (PDF) [file pone.0212769.s007.pdf]

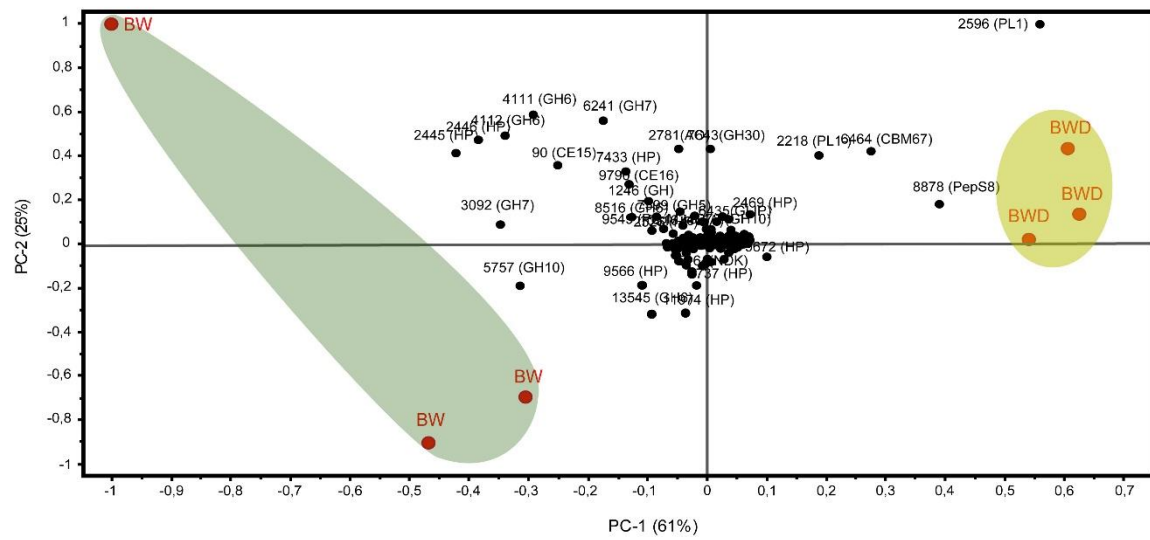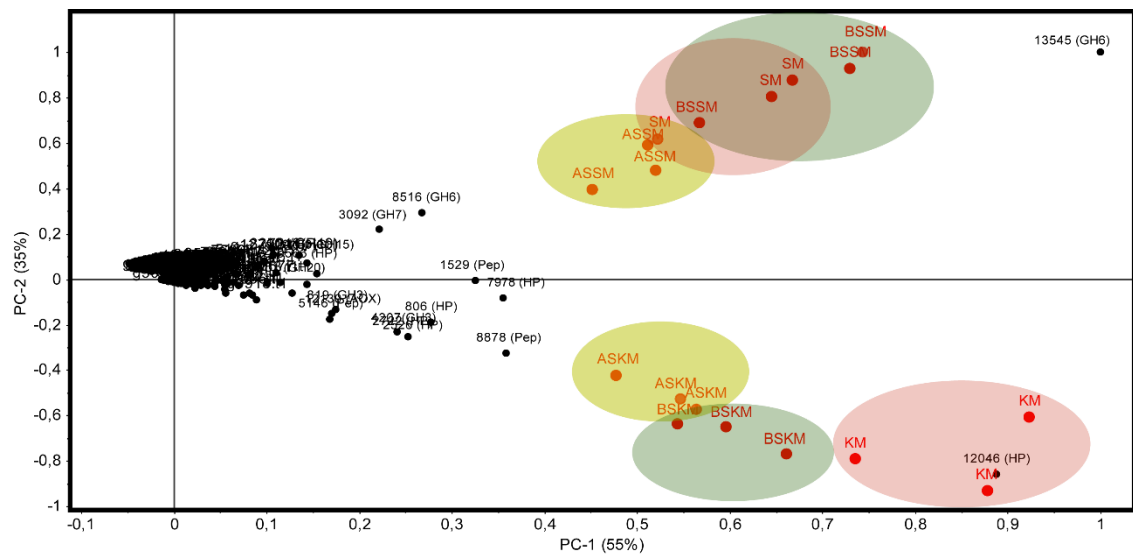

Supplement: S8 Fig — (left; BW and BWD loadings are highlighted in red) and SF cultures (right; SM, ASSM, BSSM, KM, ASKM and BSKM loadings are highlighted in red) using NIPALS algorithms. (PDF) [file pone.0212769.s008.pdf]

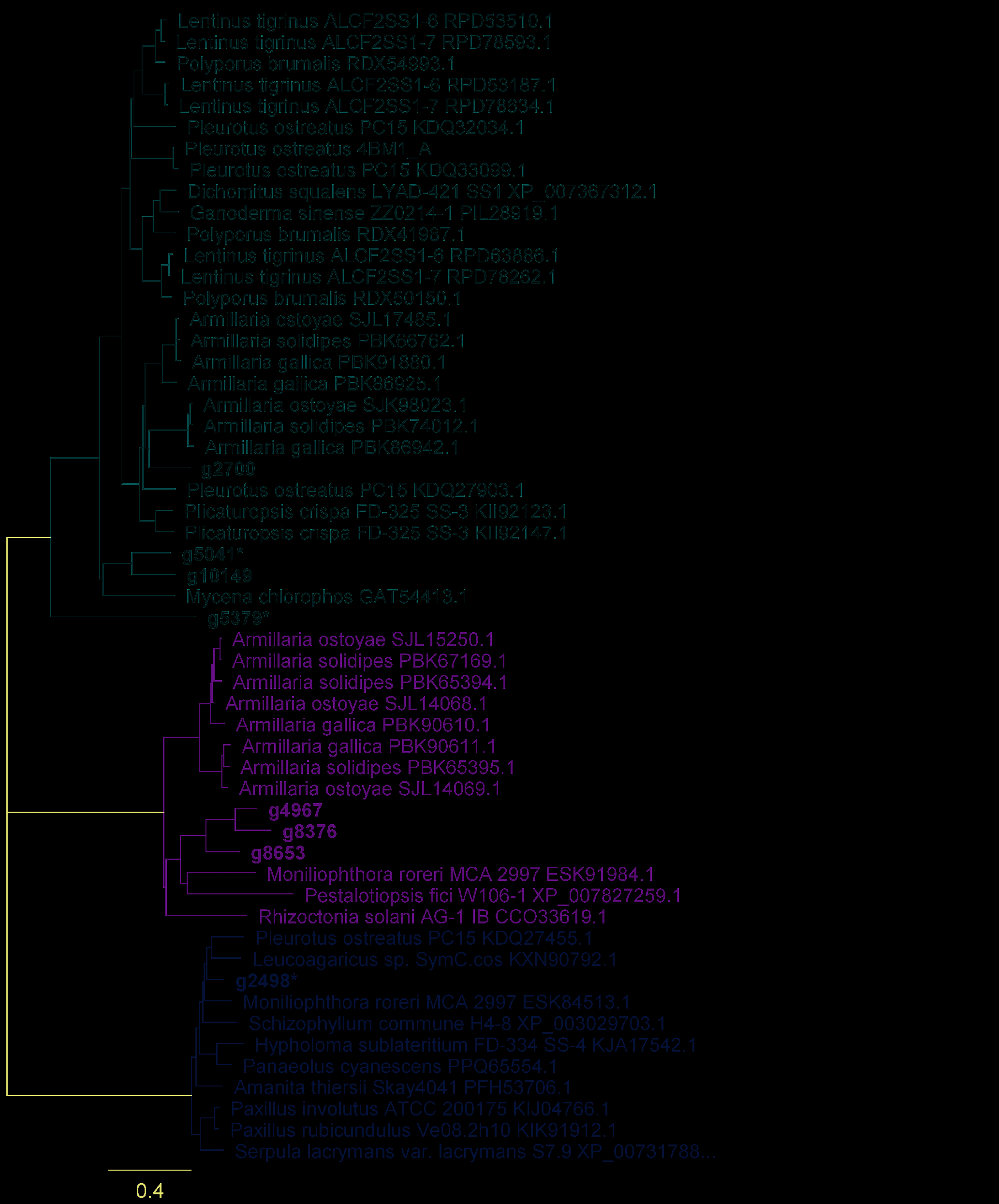

Supplement: S9 Fig — The sequences of C. purpureum are marked by a “g” and in bold. Numbers with asterisks indicate proteins detected in the secretomes; only complete sequences were considered. Sequences were aligned with Clustal W and Jukes-Cantor distance models were used. (TIF) [file pone.0212769.s009.tif]

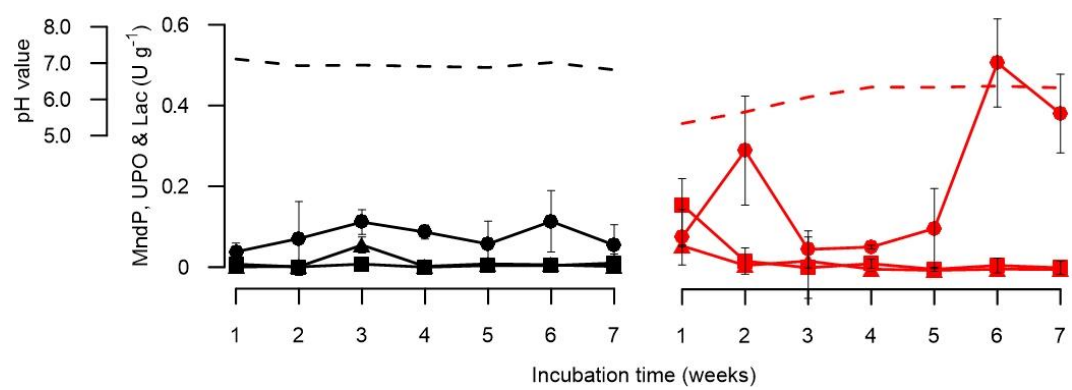

Supplement: S11 Fig — Time course of extracellular oxidoreductase production by C. purpureum during solid-state fermentation (SSF) of cultures containing beech wood (BW, left) and beech wood supplemented with olive-mill residues ‘DOR’ (BWD, right); manganese-dependent peroxidase activities (MnP, squares), unspecific peroxygenase (UPO, circles) and laccase activities (Lac, triangles) and pH (dashed line). (PDF) [file pone.0212769.s011.pdf]

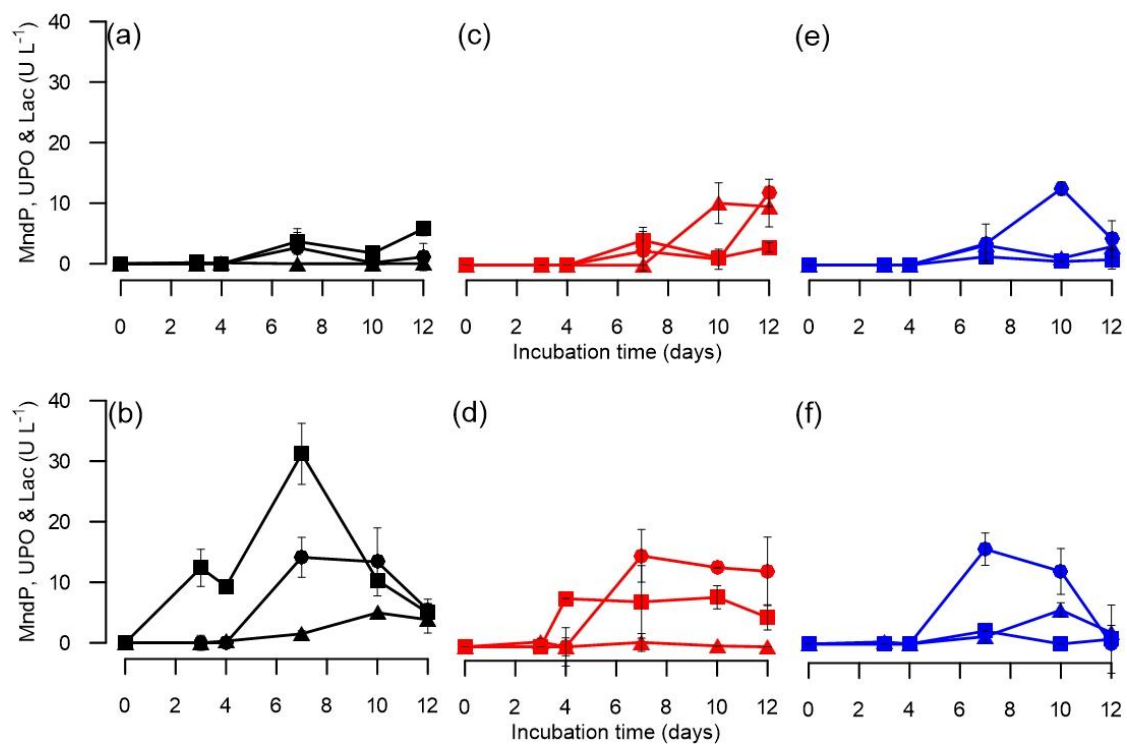

Supplement: S12 Fig — Time course of extracellular oxidoreductase production by C. purpureum during SF in cultures containing (a) Kirk medium (KM) and (b) soybean meal suspension (SM), (c) KM-ADOR (ASKM), (d) SM-ADOR (ASSM), (e) KM-birch wood (BSKM) and (f) SM-birch wood (BSSM); manganese-dependent peroxidase activities (MnP, squares), unspecific peroxygenase (UPO, circles) and laccase activities (Lac, triangles). (PDF) [file pone.0212769.s012.pdf]

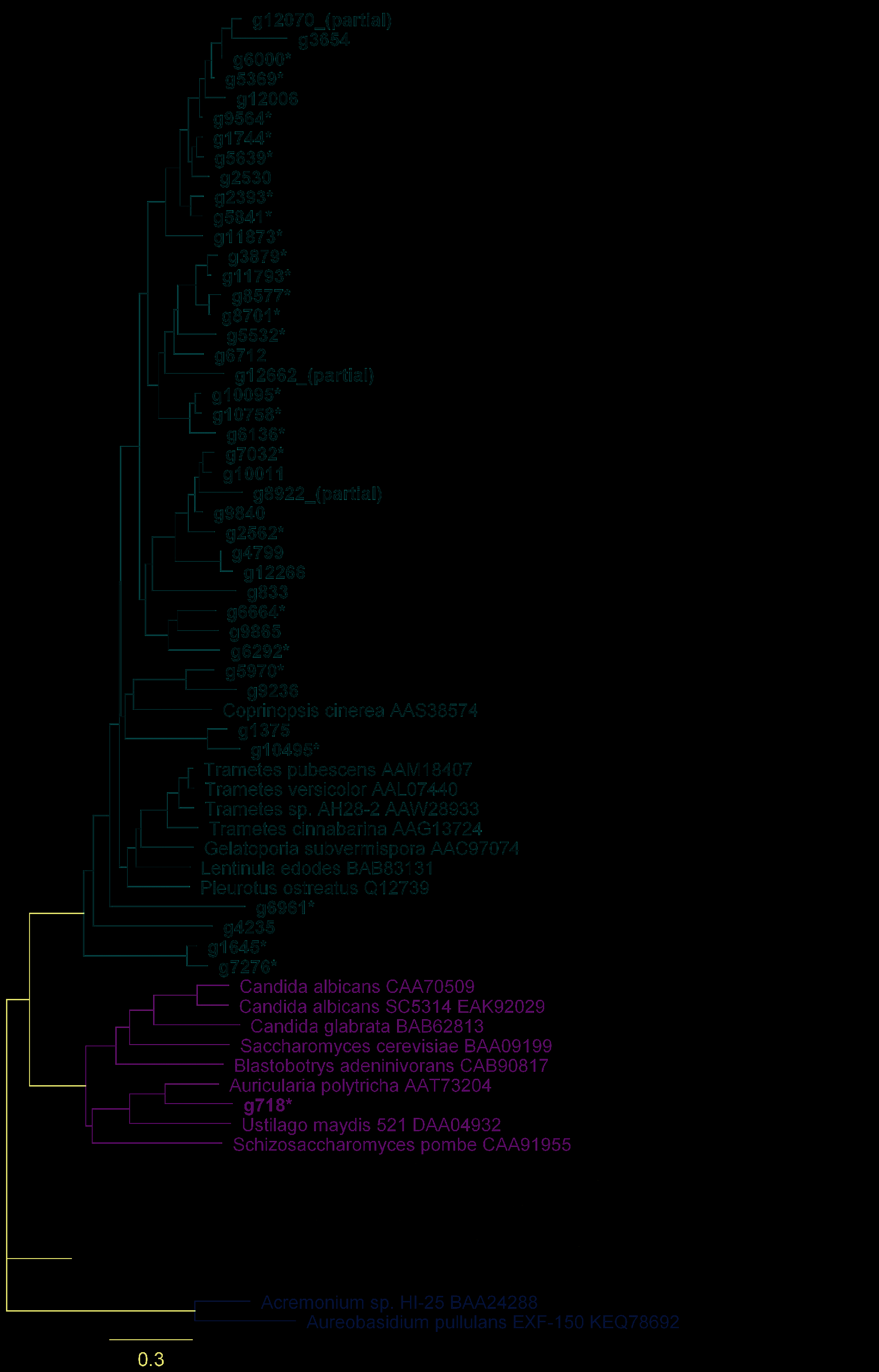

Supplement: S13 Fig — The sequences of C. purpureum (38 full length and 3 partial sequences (from C-terminal)) are marked by an “g” and in bold. Numbers with asterisks indicate that the proteins were found in the secretome. Sequences were aligned by Clustal W and Jukes-Cantor distance model were used. (TIF) [file pone.0212769.s013.tif]
