# Supplementary material for: Genome and secretome of Chondrostereum purpureum correspond to saprotrophic and phytopathogenic life styles
Source: PLoS One. 2019 Mar 1;14(3):e0212769. doi: 10.1371/journal.pone.0212769 (PMC6396904; doi:10.1371/journal.pone.0212769)
Supplement: S4 Fig — Agabi, Agaricus bisporus; Agrae, Agrocybe aegerita (Gupta et al. 2018); Armce, Armillaria cepistipes; Armga, Armillaria gallica; Armme, Armillaria mellea; Armso, Armillaria solidipes, Armos, Armillaria ostoyae; Aursu, Auricularia subglabra; Bjead, Bjerkandera adusta; Botbo, Botryobasidium botryosum; Cersu, Ceriporiopsis subvermispora; Chopu, Chondrostereum purpureum; Conpu, Coniophora puteana; Copci, Coprinopsis cinereus; Dacsp, Dacryopinax sp.; Dicsq, Dichomitus squalens; Fomme, Fomitiporia mediterranea; Fompi, Fomitopsis pinicola; Galma, Galerina marginata; Glotr, Gloeophyllum trabeum; Hetan, Heterobasidion annosum; Jaaar, Jaapia argillacea; Mycch, Mycena chlorophos (Tanaka et al. 2014); Monpe, Moniliophthora perniciosa; Phaca, Phanerochaete carnosa; Phchr, Phanerochaete chrysosporium; Phlbr, Phlebia brevispora; Pleos, Pleurotus ostreatus; Pospl, Postia placenta; Punst, Punctularia strigosozonata; Pycci, Pycnoporus cinnabarinus; Schco, Schizophyllum commune; Serla, Serpula lacrymans; Stehi, Stereum hirsutum; Trave, Trametes versicolor; Volvo, Volvariella volvacea and Wolco, Wolfiporia cocos; *generic peroxidase, ! probably a generic peroxidase. (PDF) [file pone.0212769.s004.pdf]

Order	Po								Au	Hy	Co	Ru		Ag														Ca	Ja	Ag	Po			Da	Gl	Bo			
	Bjead	Cersu	Dicsq	Phaca	Phchr	Phlbr	Pycci	Trave	Aursu	Fomme	Punst	Hetan	Stehi	Agabi	Agrae	Armce	Armga	Armme	Armso	Armos	Chopu	Copci	Galma	Mycch	Monpe	Pleos	Volvo	Botbo	Jaaar	Schco	Fompi	Pospl	Wolco	Dacsp	Glotr	Conpu	Serla		
Crystalline cellulose	32	16	17	27	30	28	20	23	48	6	21	17	17	17	30	9	13	11	11	9	11	39	54	25	5	33	56	28	24	5	0	0	0	1	1	2	8	CBM1	Carbohydrate-binding module family 1
	1	1	1	1	1	1	1	1	2	2	1	1	1	1	4	2	2	2	2	2	6	5	3	1	3	3	5	3	3	1	0	0	0	0	0	2	1	GH6	Glycoside hydrolase family 6
	5	3	4	5	8	4	3	4	6	2	5	1	3	1	11	5	4	4	3	5	9	6	8	6	5	16	11	7	5	2	0	0	0	0	0	2	0	GH7	Glycoside hydrolase family 7
	28	9	15	11	15	12	17	18	19	13	14	10	16	11	21	22	19	25	19	19	31	34	19	12	18	29	31	32	15	22	4	2	2	0	4	10	5	AA9	Lytic polysaccharide monooxygenase
Lignin	20	17	12	11	15	15	11	26	19	17	11	8	6	2	4	8	8	10	7	7	4	1	10	11	4	9	7	0	1!	0	1*	1*	1*	0	0	0	0	AA2	Class II peroxidase
	30	17	27	32	31	32	20	17	38	23	18	29	40	31	25	67	65	52	51	54	24	35	32	2	51	36	27	19	17	18	16	15	8	8	20	14	8	AA3_2	GMC oxidoreductase
	7	3	9	6	7	8	7	9	9	4	9	5	8	9	8	5	5	7	5	5	12	6	15	10	7	16	4	5	4	2	4	2	4	3	2	6	3	AA5_1	Copper radical oxidase
	0	7	11	0	0	8	5	7	0	10	12	14	15	12	14	23	25	24	23	24	45	17	8	13	24	11	11	0	1	2	5	4	3	0	4	6	4	AA1_1	Laccase
	7	4	4	4	3	6	2	4	7	3	4	3	7	5	3	8	9	8	6	6	10	2	6	18	5	4	5	3	2	4	5	1	6	1	2	5	5	AA3_3	Alcohol oxidase
	4	0	4	0	0	0	0	0	2	0	1	2	3	3	2	3	5	3	4	4	3	2	9	2	0	3	2	5	1	4	5	0	0	3	0	0	0	AA7	Glucooligosaccharide oxidase
	2	1	1	1	1	2	1	2	0	1	1	1	2	1	1	2	2	3	2	2	1	0	1	0	5	1	0	1	1	0	1	1	1	3	1	1	1	AA1_2	Ferroxidase
	1	1	1	1	1	1	1	1	1	1	1	1	1	1	0	3	3	3	3	3	1	1	1	1	3	1	1	3	1	1	0	0	0	0	1	2	2	AA3_1	Cellobiose dehydrogenase
	5	0	0	0	1	1	2	1	3	0	1	0	0	0	0	3	3	4	0	2	1	0	0	0	0	0	0	2	1	0	0	0	0	0	1	0	0	AA3_4	Pyranose oxidase
	0	0	0	0	4	0	0	0	0	1	0	1	3	1	0	0	0	1	0	0	1	0	0	0	1	0	0	0	0	4	0	0	0	2	0	1	0	AA1_dist	Multicopper oxidase
	4	0	1	3	4	4	1	1	4	3	2	1	1	4	3	3	3	3	4	4	4	3	3	0	1	2	2	1	3	4	1	0	1	1	3	2	2	AA6	Benzoquinone reductase
	1	2	2	2	2	2	2	2	1	1	1	2	2	1	1	2	2	2	2	2	2	6	1	1	2	1	1	2	2	3	0	0	0	0	0	4	4	AA8	Iron reductase domain
	0	0	0	0	0	0	0	0	0	0	0	0	0	0	0	0	0	0	0	0	3	0	0	0	0	0	0	0	2	0	0	0	0	2	3	0	0	AA4	Vanillyl alcohol oxidase
	9	0	1	3	0	3	0	2	11	3	5	1	2	2	3	1	0	2	1	1	7	4	5	1	1	5	1	3	1	1	0	2	0	0	0	0	0	DyPs	DyP-type peroxidase
	4	9	4	2	5	2	3	3	15	3	8	4	9	22	18	8	6	4	6	6	8	7	21	3	11	4	3	7	8	3	5	5	5	6	6	2	3	HTP	Heme-thiolate peroxidases
