# Supplementary material for: Genome and secretome of Chondrostereum purpureum correspond to saprotrophic and phytopathogenic life styles
Source: PLoS One. 2019 Mar 1;14(3):e0212769. doi: 10.1371/journal.pone.0212769 (PMC6396904; doi:10.1371/journal.pone.0212769)
Supplement: S16 Fig — The middle section indicates the number of orthogroups housing genes in each of the respective classes and their copy number in each species. Agabi, Agaricus bisporus; Agrae, Agrocybe aegerita; Armce, Armillaria cepistipes; Armga, Armillaria gallica; Armme, Armillaria mellea; Armso, Armillaria solidipes, Armos, Armillaria ostoyae; Aursu, Auricularia subglabra; Bjead, Bjerkandera adusta; Botbo, Botryobasidium botryosum; Cersu, Ceriporiopsis subvermispora; Chopu, Chondrostereum purpureum; Conpu, Coniophora puteana; Copci, Coprinopsis cinereus; Dacsp, Dacryopinax sp.; Dicsq, Dichomitus squalens; Fomme, Fomitiporia mediterranea; Fompi, Fomitopsis pinicola; Galma, Galerina marginata; Glotr, Gloeophyllum trabeum; Hetan, Heterobasidion annosum; Jaaar, Jaapia argillacea; Mycch, Mycena chlorophos; Monpe, Moniliophthora perniciosa; Phaca, Phanerochaete carnosa; Phchr, Phanerochaete chrysosporium; Phlbr, Phlebia brevispora; Pleos, Pleurotus ostreatus; Pospl, Postia placenta; Punst, Punctularia strigosozonata; Pycci, Pycnoporus cinnabarinus; Schco, Schizophyllum commune; Serla, Serpula lacrymans; Stehi, Stereum hirsutum; Trave, Trametes versicolor; Volvo, Volvariella volvacea and Wolco, Wolfiporia cocos. (PDF) [file pone.0212769.s016.pdf]

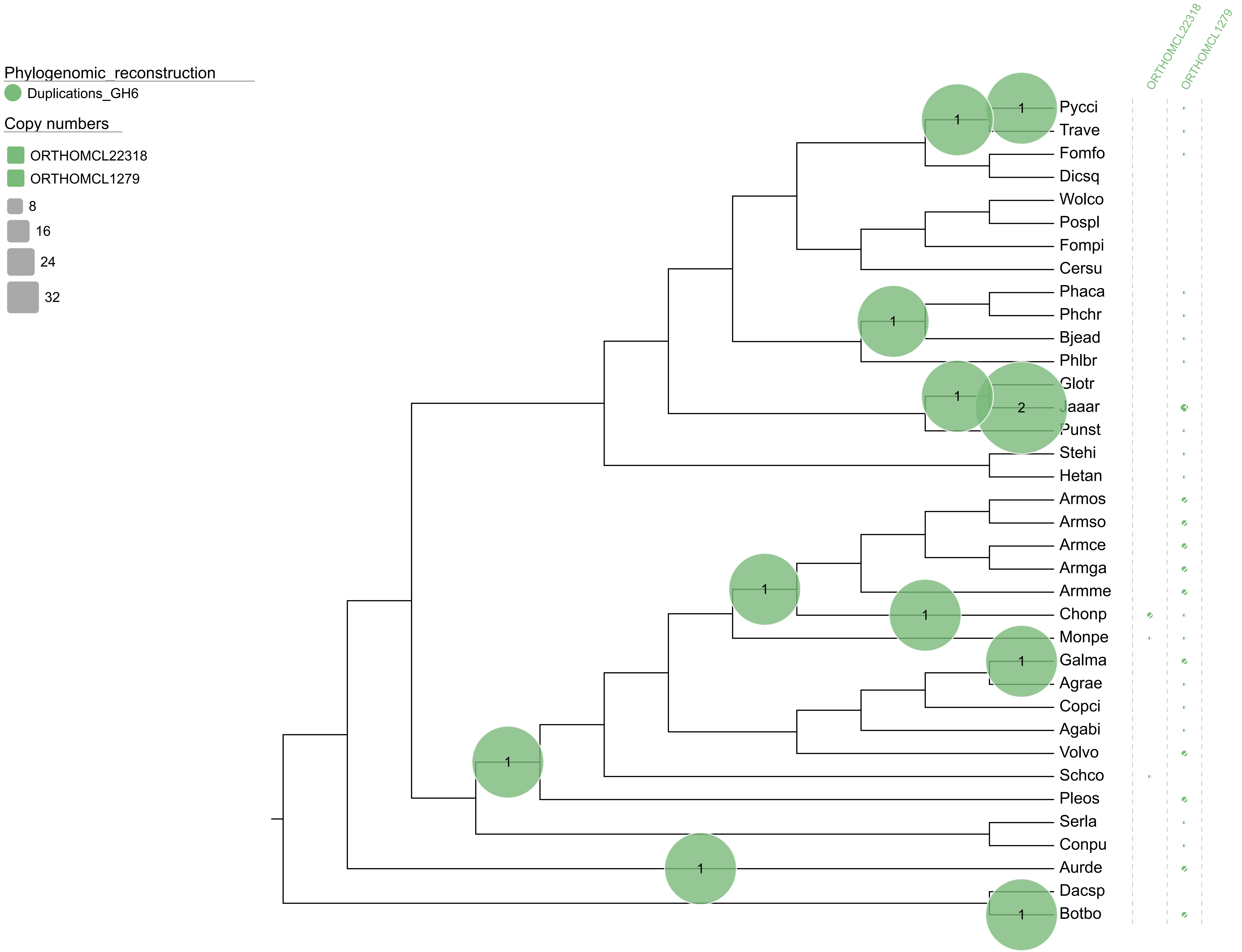
Phylogenomic_reconstruction
Duplications_GH6
Copy numbers
ORTHOMCL22318
ORTHOMCL1279
8
16
24
32
ORTHOMCL22318
ORTHOMCL1279
Pycci
Trave
Fomfo
Dicsq
Wolco
Pospl
Fompi
Cersu
Phaca
Phchr
Bjead
Phlbr
Glotr
Jaaar
Punst
Stehi
Hetan
Armos
Armso
Armce
Armga
Armme
Chonp
Monpe
Galma
Agrae
Copci
Agabi
Volvo
Schco
Pleos
Serla
Conpu
Aurde
Dacsp
Botbo
1
1
1
1
2
1
1
1
1
1
1
