# Supplementary material for: Genome and secretome of Chondrostereum purpureum correspond to saprotrophic and phytopathogenic life styles
Source: PLoS One. 2019 Mar 1;14(3):e0212769. doi: 10.1371/journal.pone.0212769 (PMC6396904; doi:10.1371/journal.pone.0212769)
Supplement: S15 Fig — Maximum Likelihood phylogeny based on 609 genes across 36 genomes. Numbers indicate bootstrap support at each node. (PDF) [file pone.0212769.s015.pdf]

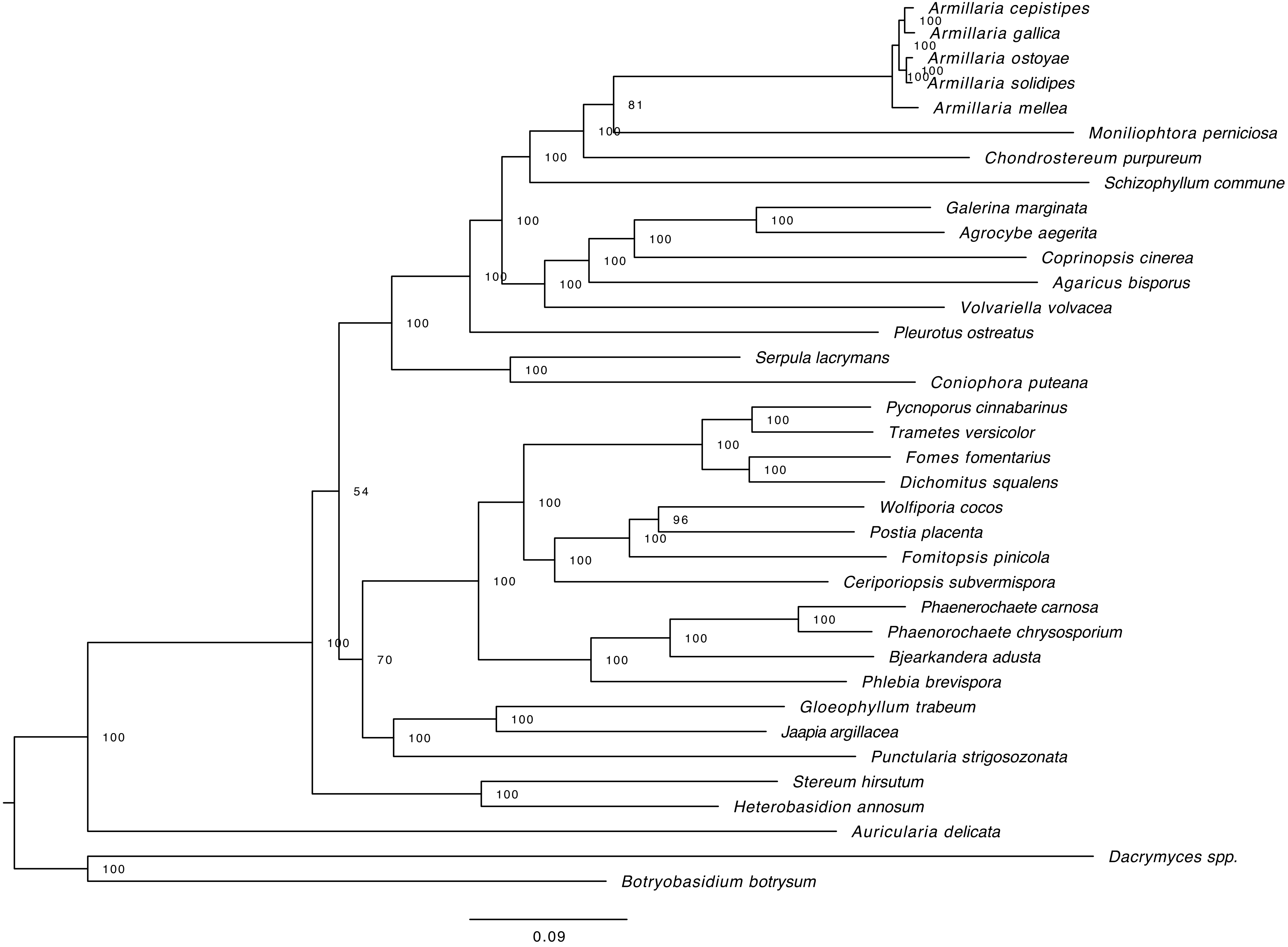
Armillaria cepistipes
Armillaria gallica
Armillaria ostoyae
Armillaria solidipes
Armillaria mellea
Moniliophtora perniciosa
Chondrostereum purpureum
Schizophyllum commune
Galerina marginata
Agrocybe aegerita
Coprinopsis cinerea
Agaricus bisporus
Volvariella volvacea
Pleurotus ostreatus
Serpula lacrymans
Coniophora puteana
Pycnoporus cinnabarinus
Trametes versicolor
Fomes fomentarius
Dichomitus squalens
Wolfiporia cocos
Postia placenta
Fomitopsis pinicola
Ceriporiopsis subvermispora
Phaenerochaete carnosa
Phaenorochaete chrysosporium
Bjearkandera adusta
Phlebia brevispora
Gloeophyllum trabeum
Jaapia argillacea
Punctularia strigosozonata
Stereum hirsutum
Heterobasidion annosum
Auricularia delicata
Dacrymyces spp.
Botryobasidium botrysum
100
81
54
70
96
0.09

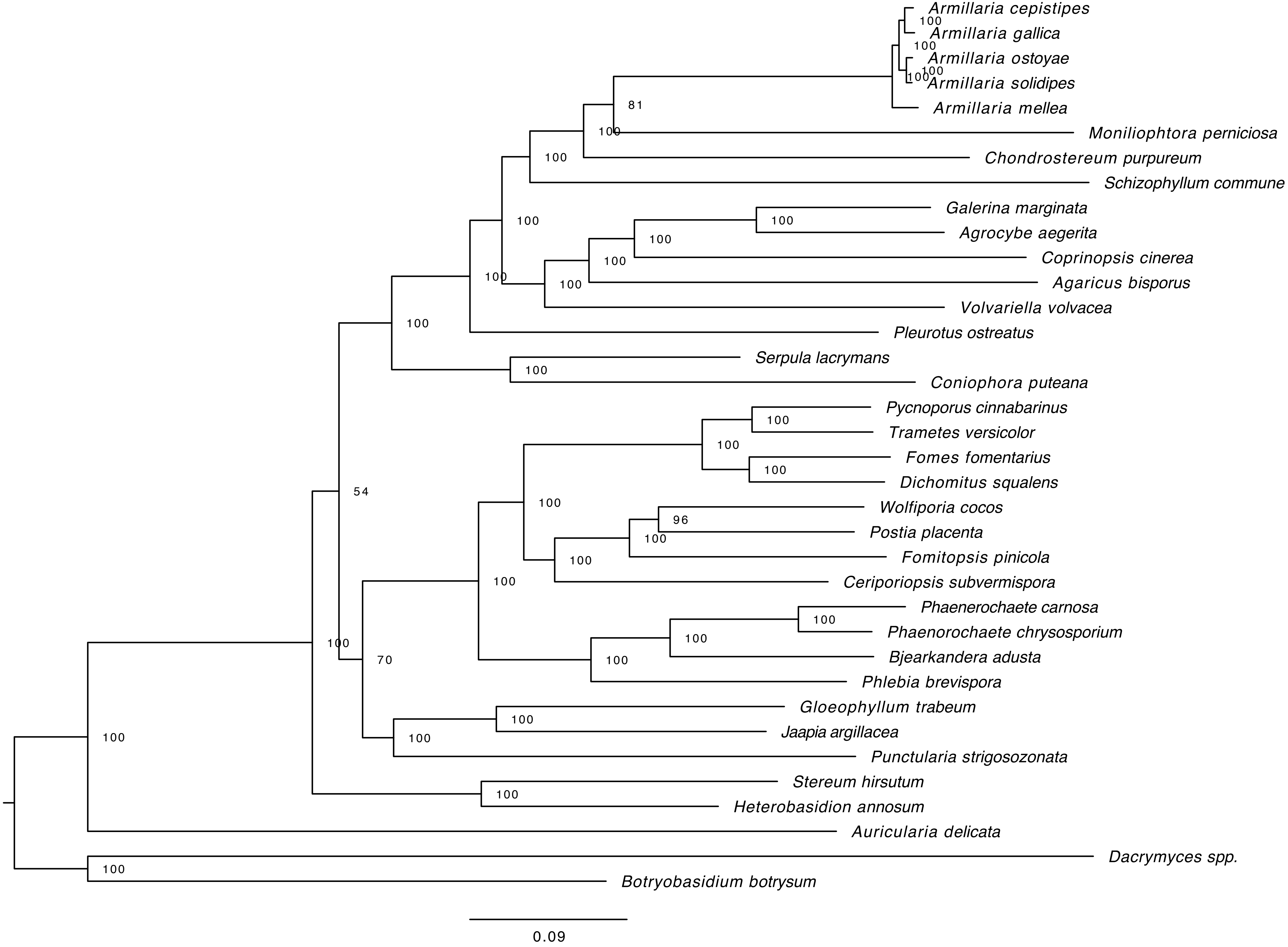
Armillaria cepistipes
Armillaria gallica
Armillaria ostoyae
Armillaria solidipes
Armillaria mellea
Moniliophtora perniciosa
Chondrostereum purpureum
Schizophyllum commune
Galerina marginata
Agrocybe aegerita
Coprinopsis cinerea
Agaricus bisporus
Volvariella volvacea
Pleurotus ostreatus
Serpula lacrymans
Coniophora puteana
Pycnoporus cinnabarinus
Trametes versicolor
Fomes fomentarius
Dichomitus squalens
Wolfiporia cocos
Postia placenta
Fomitopsis pinicola
Ceriporiopsis subvermispora
Phaenerochaete carnosa
Phaenorochaete chrysosporium
Bjearkandera adusta
Phlebia brevispora
Gloeophyllum trabeum
Jaapia argillacea
Punctularia strigosozonata
Stereum hirsutum
Heterobasidion annosum
Auricularia delicata
Dacrymyces spp.
Botryobasidium botrysum
100
81
54
70
96
0.09
